# Synthesis, Pharmacokinetic Characterization and Antioxidant Capacity of Carotenoid Succinates and Their Melatonin Conjugates

**DOI:** 10.3390/molecules27154822

**Published:** 2022-07-28

**Authors:** Dalma Czett, Katalin Böddi, Veronika Nagy, Anikó Takátsy, József Deli, Paul Tone, György T. Balogh, Anna Vincze, Attila Agócs

**Affiliations:** 1 Department of Biochemistry and Medical Chemistry, Medical School, University of Pécs, Szigeti út 12, H-7624 Pécs, Hungary; czett.dalma@gmail.com (D.C.); katalin.boddi@aok.pte.hu (K.B.); vera.nagy@aok.pte.hu (V.N.); aniko.takatsy@aok.pte.hu (A.T.); jozsef.deli@aok.pte.hu (J.D.); 2 Department of Pharmacognosy, Faculty of Pharmacy, University of Pécs, Rókus u. 2, H-7624 Pécs, Hungary; 3 Department of Medicine, Richmond University Medical Center, Staten Island, NY 10310, USA; paultonemd@gmail.com; 4 Department of Chemical and Environmental Process Engineering, Budapest University of Technology and Economics, Műegyetem rkp. 3, H-1111 Budapest, Hungary; balogh.gyorgy@vbk.bme.hu (G.T.B.); vincze.anna@edu.bme.hu (A.V.); 5 Institute of Pharmacodynamics and Biopharmacy, Faculty of Pharmacy, University of Szeged, Eötvös u. 6, H-6720 Szeged, Hungary

**Keywords:** carotenoids, succinates, melatonin, hydrophilic, lipophilic, aggregation, antioxidant, bifunctional conjugates, oxidative stress

## Abstract

Carotenoid succinates were synthesized from hydroxy carotenoids and were coupled to a commercially available derivative of melatonin via amide bond for producing more powerful anti-oxidants and yet new hybrid lipophilic bifunctional molecules with additional therapeutic effects. The coupling reactions produced conjugates in acceptable to good yields. Succinylation increased the water solubility of the carotenoids, while the conjugation with melatonin resulted in more lipophilic derivatives. The conjugates showed self-assembly in aqueous medium and yielded relatively stable colloidal solutions in phosphate-buffered saline. Antioxidant behavior was measured with ABTS and the FRAP methods for the carotenoids, the carotenoid succinates, and the conjugates with melatonin. A strong dependence on the quality of the solvent was observed. TEAC values of the new derivatives in phosphate-buffered saline were found to be comparable to or higher than those of parent carotenoids, however, synergism was observed only in FRAP assays.

## 1. Introduction

Carotenoids are known as effective antioxidants with elevated hydrophobic character and as an important part of human nutrition. They can incorporate into cell membranes and can play a role in the inhibition of oxidative stress [1]. Simultaneously, carotenoids could serve as membrane anchor in covalent conjugation with hydrophilic molecules.

Human studies have demonstrated that lutein and zeaxanthin are present in the skin, and animal studies have provided evidence of their significant efficacy against light-induced skin damage, especially the ultraviolet wavelengths, by acting as antioxidants to protect against the formation of reactive oxygen species and subsequent free radicals [2]. Supplementation with lutein/zeaxanthin improves central nervous system xanthophylls levels and cognitive function in young healthy adults [3], older men and women [4]. Zeaxanthin and lutein were also established being protective agents against age-related macular degeneration [5]. It is believed, based on data derived from human interventions, animal models of research and cell studies, that zeaxanthin exerts its antioxidant protection on eye, arteries, blood, skin and liver [6]. Only a little is known about the physiological effects of κ-carotenoids, such as capsanthin, a main pigment of paprika and some other red fruits. However, κ-carotenoids were proven to be excellent antioxidants in vitro [7]. β-cryptoxanthin has a provitamin-A structure and is an important dietary source of retinol. Additionally, β-cryptoxanthin seems to play an important role in bone formation [8]. To our knowledge the non-antioxidant properties of carotenoids and their metabolites i.e., participation in cell signaling can be as important as their direct antioxidant effect [9].

Melatonin as a hormone regulates most importantly the circadian rhythm, but is one of the best natural antioxidants and radical scavengers, as well. Besides its direct effects, melatonin triggers signal transduction through melatonin receptors [10]. It promotes the expression of antioxidant enzymes such as superoxide dismutase, glutathione peroxidase, glutathione reductase, and catalase [11]. It affects the immune system, as well, probably acts as an anti-inflammatory agent [12]. There is growing evidence that melatonin has a role in the prevention of various diseases such as cancer [13], and can even attenuate conditions caused by virus SARS-CoV-2, which induces COVID-19 [14]. Due to its participation in cellular signaling melatonin was found to be a treatment possibility in various neurodegenerative diseases such as Parkinson’s and Alzheimer’s [15]. The pleiotropic effect of melatonin can be further improved by covalent derivatization, as some examples have already demonstrated [16,17,18,19].

From a molecular point of view, melatonin is a small size amphiphilic substance. For this reason, it is fairly soluble both in fats [20] and in water [21] preferentially located at hydrophilic/hydrophobic interfaces, and can easily cross all the anatomic barriers and distribute throughout the cell [22]. Amphiphilicity of exogenous and endogenous melatonin enable this molecule not only to cross any cell membrane into the cytoplasm, and enter all subcellular cytosolic, mitochondrial, and nuclear compartments but also to cross the blood-brain barrier and blood-retinal barrier [23].

In this work we present the covalent coupling of carotenoids with melatonin. The carotenoid-melatonin conjugates are expected to counteract oxidative stress directly and indirectly. The conjugation is assumed to improve the cell membrane penetration of the carotenoid due to melatonin moiety, hence these hybrid bifunctional molecules could show enhanced intracellular antioxidant activity. Anti-inflammatory, immune modulatory, oncostatic effects of the conjugates can be postulated, as well.

For the conjugation, an amino derivative of melatonin was planned to couple with succinates of diverse carotenoids [24]. Carotenoid succinates are stable crystalline compounds in which the free carboxyl group offers new ways of functionalization compared to hydroxy carotenoids [25,26]. They can be produced in a straightforward reaction practically from any hydroxy carotenoid in good yields, and in a pure crystalline form [27,28,29]. Among them specifically astaxanthin disuccinate was found to be an efficient anti-inflammatory and cardioprotective agent, its disodium salt (Cardax^TM^) is a commercially available product [30,31].

Here, the in silico and In vitro physicochemical and pharmacokinetic characterization and the antioxidant capacity of the newly synthetized carotenoid conjugates were also described.

## 2. Results and Discussion

### 2.1. Synthesis

A C_30_ apocarotenoid with a primary hydroxyl group (8′-apo-β-carotenol, **1**), cryptoxanthin (**3**) bearing an OH only on one end group, lutein (**5**) and zeaxanthin (**2**), as well as the dihydroxy κ-carotenoid, capsanthin (**4**) were chosen for the conjugation with melatonin. In the case of zeaxanthin coupling with one or two melatonin moieties were examined, as well.

An amino derivative of melatonin, 2-amino-*N*-[2-(methoxy-1*H*-indol-3-yl]ethyl)-acetamide hydrochloride (**12**) is commercially available. To prepare the conjugates, carotenoid succinates were synthetised first [24], and as starting material, they were coupled with the amino-melatonin via amide bond. Primary and secondary hydroxyl groups of carotenoids, also in allylic position, were acylated by succinic anhydride in the presence of DMAP (*N*,*N*-dimethylaminopyridine) (Table 1). The prepared succinates are more hydrophilic than the parent carotenoids, and can show enhanced biological activity themselves, such as astaxanthin disuccinate [30,31]. On the other hand esters or amides can be synthetized from them relatively easily [26]. Previously we used them for the synthesis of hydrophilic carotenoids and dendrimers [25,32]. Here we give their complete analytical and spectroscopical description (Appendix A—Experimental Section).

We planned the preparation of zeaxanthin conjugates both with one and two melatonin moieties. Zeaxanthin monosuccinate (**11**) can be obtained from zeaxanthin as main product if only 1.5 eq succinic anhydride is added in 3 portions with 3 h time difference. The monosuccinate proved to be difficult to isolate and separate from bissuccinate, hence we used the reaction mixture having approx. 3:2 monosuccinate:bissuccinate ratio for the subsequent coupling.

**Figure 1:**
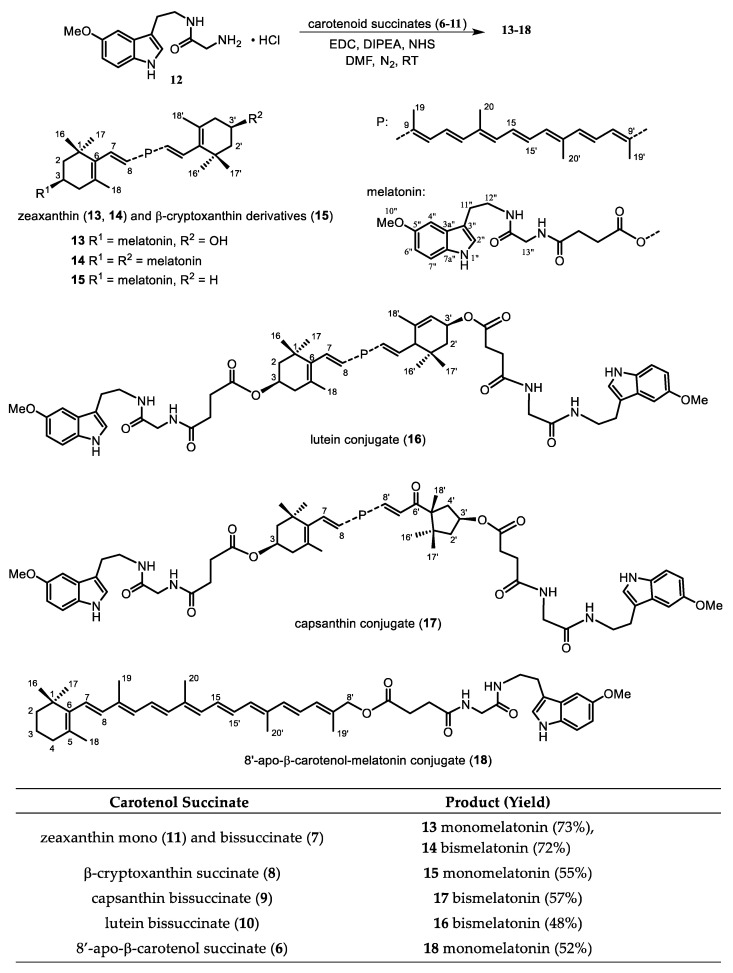
Coupling Reaction of Carotenol Succinates with Amino-melatonin.

There are plenty of methods for amide coupling in the literature, we decided not to apply the well established Steglich method using DCC/DMAP because of possible purification problems. A good alternative for peptide synthesis and for larger biomolecules is EDC (1-ethyl-3-(3-dimethylaminopropyl)carbodiimide), also in combination with NHS (*N*-hydroxy-succinimide). As a basic catalyst diisopropylethyl amine (DIPEA) was used, also to liberate EDC and the amino-melatonin **12** from their corresponding hydrochloride salts. After optimization of the conditions and the order of the reagents added the reaction provided the target compounds with acceptable yields after column chromatography and crystallization (Figure 1).

In some cases subsequent separation by preparative TLC was necessary. Starting from the zeaxanthin mono- and bissuccinate mixture the reaction delivered approximately the same amount of the mono- and bismelatonin conjugates, which could be separated and characterized (Appendix A—Experimental Section).

UV spectra of succinates and adducts was practically the same as those of the parent carotenoids. NMR spectra were practically the addition of the signals of the carotenoid and the melatonin. Significant new signals were the protons of the succinate around 2.5 ppm and the appearance of two amide bonds in the adducts (169 and 172 ppm in the ^13^C-NMR).

### 2.2. Physicochemical Characterization of Investigated Carotenol Derivatives

#### 2.2.1. In Silico and in Vitro Physicochemical and Pharmacokinetic Characterization of Carotenol Derivatives

For the characterization of the new caretenol conjugates, in silico parameters were chosen to evaluate their drug-likeness properties according to Lipinski Ro5 [33] and early-phase ADME properties of the compounds. Based on the predicted parameters given in Table 2, it can be seen that the conjugates are excluded from the chemical space designated by Ro5 in terms of both their average molecular weight and their lipophilicity. The elevated lipophilicity value (logP ≫ 5) of both carotenols (**1**–**5**) and their conjugates (**6**–1**8**) is well seen in the predicted values. It can also be seen that for succinate conjugates (**6**–**11**), logD_7.4_ values are lower than expected. In parallel, it can be seen that higher estimated aqueous solubility data (Aq.Sol.) were obtained in this range of compounds on average.

Similarly, based on the experimental kinetic solubility data, we were able to provide solubility data only for the **6**–**8** succinate conjugates, and for the other carotenol derivatives we found solubility conditions below the limit of detection (LOD, see Table 4). The best solubility value was obtained for the underivatized melatonin. For the 8′-apo-β-carotenol succinate (**6**), the highest measurable kinetic solubility can be associated with the relatively low molecular weight. In the test group, the TPSA values for the basic carotenol derivatives (**1**–**5**) and melatonin are below 120 Å^2^, which also meets the oral absorption rule and the blood-brain barrier (BBB) absorption criterion (TPSA < 90 Å^2^) [37]. In contrast, higher TPSA values were obtained for both succinate and melatonin conjugates. Only derivatives **6**, **8** (TPSA = 63.6 Å^2^) and **15**, **18** (TPSA = 109.5 Å^2^) were exceptions. Closely related to the basic physicochemical parameters, the predicted Caco-2 permeability values of the caretenol compounds were also found to be low. Furthermore, their expected brain penetration and logBB values were also decreased. Based on the data, it can be stated that in the case of 8′-apo-β-carotenol (**1**) and of **6**, **7**, **9**, **10** succinate conjugates we could expect low intestinal absorption, and in the case of melatonin an increased Caco-2 Pe value was indicated by a prediction method. In parallel with the low estimated TPSA values, prediction **1**–**6** and melatonin indicated low to medium logBB values, while inhibited brain penetration was expected for succinate and melatonin conjugates based on logBB = −2 values. Our experimental data showed a good agreement with the in silico data, as already shown by the kinetic solubility data. Unfortunately, due to poor aqueous solubility and increased lipophilicity, we were only able to give the in vitro brain lipid specific permeability value for melatonin, which was determined by our previously developed BBB-PAMPA method [38]. The low Pe value of melatonin is in good agreement with the predicted low logBB value. Overall, the unfavorable physicochemical properties of the caretenol conjugates suggest the development of a well-designed formulation strategy due to their expected beneficial biological effects, and the direct administration of these compounds is also challenging due to their solubility and permeability properties.

#### 2.2.2. Aggregation

It has been known for a long while that carotenoids or their derivatives can be prone to supramolecular organization in water or water-organic solvent mixtures [39]. This behaviour is especially interesting because this higher organization can modulate or alter biological properties of carotenoids, as well [40]. A tendency to form supramolecular structures can be tested by recording the UV spectra in an organic solvent and increasing the ratio of water in the sample. Based on if and how the spectrum changes one can suggest the formation of J- or H-type aggregates, although other spectroscopic methods such as eg. circular dichroism is needed for confirmation [41,42]. We have some recent experience of such behaviour of carotenoid-flavonoid conjugates [43].

All of our adducts were tested with the method above using tetrahydrofurane (THF) or dimethylsulfoxide (DMSO) with increasing ratio of phosphate-buffered saline (PBS), which was the medium of the antioxidant measurements. Starting from THF solutions, the increasing ratio of PBS did not cause much change either in the spectra of bissuccinates (**7**, **9**, **10**) or in the spectra of both derivatives of 8′-apo-β-carotenal (**6**, **18**). However, all the other melatonin conjugates (**13**, **14**, **15**, **16**, **17**) and the monosuccinates of zeaxanthin (**11**) and β-cryproxanthin (**8**) showed a bathochromic shift in their UV spectra recorded in THF:aqueous solutions compared to that in pure THF (Figures S1–S12). That suggests the propensity of these compounds (**8**, **11**, **13**, **14**, **15**, **16**, **17**) to form J-type (head-to-tail) aggregates from THF.

In contrary, all spectra gave a hypsochromic shift and loss of fine-structure when the DMSO solutions were diluted with PBS, which refers to the formation of H-type (card-pack) aggregates (Figures S1–S12). Since DMSO-containing aqueous systems are more suitable for biological studies, we examined these solution by dynamic light scattering (DLS) spectroscopy to gain more information about the particles. The concentrations of the examined solutions were the same as used in the ABTS/TEAC measurements (0.3125–5.00 μM, see next section).

**Scheme 1:**
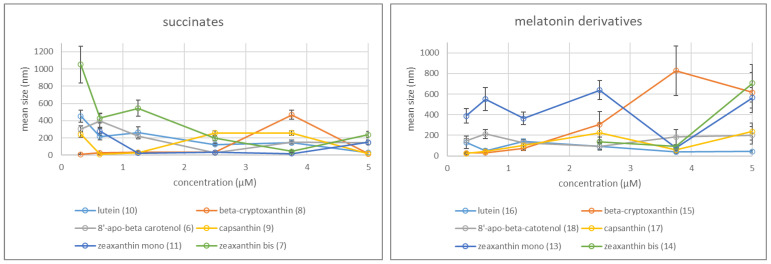
Average Size (Hydrodynamic Diameter) of the Aggegates Determined by DLS at Different Concentrations.

The hydrodynamic diameter of the succinate aggregates varies typically between 10 and ~400 nm (Scheme 1). Bissuccinates of lutein (**10**) and zeaxanthin (**7**), as well as 8′-apo-β-carotenol monosuccinate (**6**) paradoxically tend to form larger aggregates at lower concentrations. That might be due to the different DMSO content (0.25–2%) of the final solutions. In the case of the monosuccinates of β-cryptoxanthin (**8**) and zeaxantin (**11**), however, the size of the particles hardly depends on the concentration. Interestingly, only at certain concentrations (3.75 μM for **8**, 5 μM and 0.625 μM for **11**) these molecules assemble to huge particles (approx. a tenfold size compared to those at other concentrations).

The melatonin derivatives generally form aggregates between 10–200 nm (Scheme 1). The exceptions are zeaxanthin monomelatonin conjugate (**13**) forming generally 400–600 nm size particles, and β-cryptoxanthin-melatonin (**15**), that shows a strong concentration dependence. The bismelatonin conjugate of zeaxanthin (**14**) gave so large particles at low concentrations, that their size could not be determined by DLS.

On the basis of the ζ potentials, most of the prepared solutions proved to be relatively stable colloidal systems. With the exception of 8′-apo-β-carotenol monosuccinate (ζ = −10–−30 mV), all the succinates showed high positive ζ potential values (>20 mV), which, however, depended on the concentration. The melatonin conjugates gave negative ζ potential values, but only the derivatives of 8′-apo-β-carotenol (**18**), β-cryptoxanthin (**15**), and capsanthin (**17**) (ζ = −20–−30 mV) seemed to be relatively stable at each concentration. For the experimental data see the Supplementary Materials (pp. 6–8).

As the prepared succinate solutions were mostly stable colloids, it encouraged us to determine experimentally the water dispersibility of the newly synthetised carotenoid succinates. Coupling of carotenols with succinic acid highly increased their water dispersibility compared to the native carotenoids (Table 3). However, further conjugation with melatonin made the products insoluble in water, and increased the lipophilicity of melatonin.

### 2.3. Antioxidant Properties

Antioxidant measurements were made with ABTS/TEAC [44,45] and with the FRAP assays [46] to estimate changes in the antioxidant capacity compared to the carotenoids and underivatized melatonin. In both methods we had to modify the literature procedure slightly to obtain more reliable results. For a statistical evaluation, please, see the Supplementary Materials (pp. 9–11).

#### 2.3.1. ABTS/TEAC Assays

**Scheme 2:**
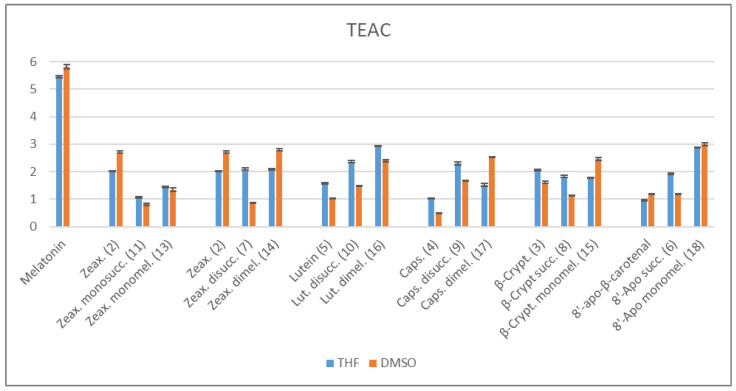
TEAC values determined using ABTS^•+^ radical (*p* < 0.05).

This method uses a redox reaction between ABTS^•+^ radical cation and the examined antioxidants, which act as the reducing agents. The TEAC values were measured for the starting carotenoids, their succinates, for melatonin and the adducts according to a standard procedure [44,45]. Note, that the parent 8′-apocarotenol can not be used for reference, since it is very susceptible for oxidation, thus the stable 8’-apocarotenal was used instead. As the carotenoids and the conjugates are poorly soluble in water, they were dissolved first either in small amounts of freshly distilled tetrahydrofuran (THF) or in dimethylsulfoxide (DMSO), and these stock solutions were diluted with phosphate-buffered saline (PBS) to the final nominal concentration. The results are summarized in the diagram below (Scheme 2).

It is conspicuous that the TEAC values depend on the quality of the initial solvent, even though it is present in very small amounts in the final solution (0.25–2%). As the aqueous medium induces self-organization of the carotenoid derivatives, the determined TEAC values most probably do not belong to the individual molecules but to their aggregates. As we described it in the previous section, THF and DMSO conduct the formation of different type aggregate structures.

For the monoacylated zeaxanthin and diacylated lutein, the changes of TEAC values compared to the native carotenoids show similar tendencies independently from the initial solvent. In the case of zeaxanthin, acylation with one succinate and further conjugation with one melatonin led to less active derivatives than the parent zeaxanthin. In contrary, succinylation and further coupling with melatonin resulted in considerable increase of TEAC values starting from lutein.

When the stock solution was prepared with THF, zeaxanthin gave the same TEAC values as its bissucinate and the bismelatonin conjugate. From DMSO, however, zeaxanthin bissuccinate showed an unexpectedly low antioxidant activity. Derivatives of β-cryptoxanthin also showed similar TEAC as the parent carotenoid from THF, while from DMSO stock solution the activity of β-cryptoxanthin succinate decreased, that of its melatonin conjugate increased significantly.

All derivatives of capsanthin and 8′-apo-β-carotenol displayed higher antioxidant activity than the corresponding native carotenoids, but their relative order depends on the initial solvent. From THF, capsanthin bissuccinate had the highest TEAC value, while from DMSO, its bismelatonin conjugate proved to be the most active. Similarly to the case of zeaxanthin bissuccinate, the 8′-apo-β-carotenol succinate also showed a relatively low TEAC from DMSO compared to the tendency measured from THF.

Hydrophilic derivatives of carotenoids, such as succinates, are known to display an elevated antioxidant efficiency in an aqueous medium compared to their hydrophobic parent compounds [47]. Interestingly, in our experiments the even more hydrophobic melatonin conjugates of lutein, capsanthin and 8′-apo-β-carotenal also exhibited outstanding TEAC values. The hydrodynamic diameter of the aggregates of these melatonin conjugates (**16**, **17**, **18**) showed the smallest change in the function of concentration (see Scheme 1 and the Supplementary Materials pp. 6–8).

Here is to be noted that the behaviour of the two isomers, lutein and zeaxanthin, and their derivatives were rather different. To examine the effect of the covalent coupling, we also measured the antioxidant behaviour of a zeaxanthin:melatonin 1:2 mixture, which gave enormous TEAC values (12.04 from THF and 13.72 from DMSO). It suggests a synergism, which seems to be lost by the covalent conjugation.

#### 2.3.2. FRAP Method

**Scheme 3:**
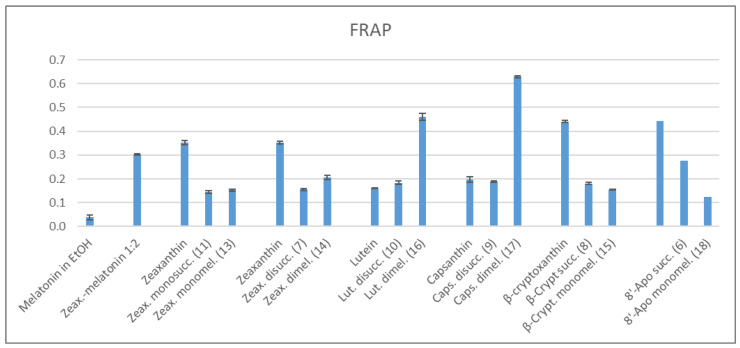
FRAP Values Measured Using Fe^III^-TPTZ in Acetate Buffer (*p* < 0.05).

In the FRAP method the antioxidant reduces Fe^3+^ to Fe^2+^ in an acidic medium [46]. These experiments were performed in an acetate buffer of pH 3.0, the stock solutions of the examined antioxidants were prepared in THF. The antioxidant capacities were compared to that of Trolox as a standard, just like in the ABTS assays. Under these conditions, melatonin showed an extremely low antioxidant capacity (Scheme 3).

When comparing the FRAP values, those of the melatonin conjugates of lutein and capsanthin were outliers, surpassing all the native carotenoids. In these cases the increase in the antioxidant capacity seems to show a synergism of melatonin and the carotenoid moieties. While the parent zeaxanthin, β-cryptoxanthin, and 8′-apo-β-carotenal showed relatively high FRAP, their succinates or melatonin derivatives fell behind. The succinates of all carotenoids had a similar FRAP values, the only exception was the 8′-apo-β-carotenol succinate with a 1.5–2 times more elevated value. Besides the above mentioned melatonin conjugates of lutein and capsanthin, the melatonin derivatives of the other carotenoids gave comparable FRAP values. Similarly to the ABTS assay, the zeaxanthin dimelatonin adduct behaves rather differently than its isomer, the lutein derivative.

## 3. Materials and Methods

The carotenoids were isolated from red pepper *Capsicuum anuum* using a well-established procedure [48]. Crude 8′-apo-β-carotenol was freshly prepared from commercially available 8′-apo-β-carotenal (Fluka), because the alcohol is susceptible to oxidation [49]. The amino-melatonin (2-amino-*N*-[2-(methoxy-1*H*-indol-3-yl]ethyl)-acetamide hydrochloride was purchased from Matrix Scientific, Columbia, SC, USA.

All reagents used for synthesis were analytically pure quality and all organic solvents were of HPLC grade. Organic solutions were dried over anhydrous Na_2_SO_4_ and concentrated in vacuo at 40 °C (bath temperature).

For kinetic solubility study, analytical grade solvents like DMSO, acetonitrile (AcN), hexane, dodecane as well as phosphate buffered saline powder (PBS) and cholesterol were purchased from Merck kGaA (Darmstadt, Germany). For blood-brain barrier-specific PAMPA measurements, Brain Polar Lipid Extract was purchased from Avanti Polar Lipids Inc., (Alabaster, AL, USA).

For the antioxidant assays the carotenoids or the conjugates were dissolved in freshly distilled tetrahydrofuran (THF) or in DMSO (VWR International Kft., Debrecen, Hungary). For the Trolox Equivalent Antioxidant Capacity (TEAC) assay the following reagents were used: 2,2′-azino-di-(3-ethylbenzthiazoline sulfonic acid) (Tokyo Chemical Industries, Tokyo, Japan), potassium persulphate (Alfa Aesar, Tewksbury, MA, USA), Gibco Dulbecco’s Phosphate-Buffered Saline (DPBS) powder without Ca^2+^ and Mg^2+^ (VWR International Kft., Hungary), trolox (Acros), melatonin (Alfa Aesar). For the Ferric Reducing Antioxidant Power (FRAP) method FeCl_3_·6H_2_O (Molar Ltd., Halásztelek, Hungary), 2,4,6-tris(2-pyridyl-*s*-triazin) (Acros, Pittsburgh, PA, USA) were used. The spectrophotometric measurements were implemented on a Jasco spectrophotometer model V-550 UV/Vis. An Elmasonic S 60 H ultrasonic apparatus was used in the solubility experiments.

### 3.1. Determination of Kinetic Solubility

For kinetic solubility studies each sample was dissolved in DMSO using ultrasonic bath for 15 min to make 10 mM stock solutions. On a 96-well polypropylene plate (Greiner bio-one) 7.5 μL stock solutions were added to 292.5 μL PBS (pH 7.4) to make a solution with the nominal concentration of 250 μM. For each sample 3 replicates were measured. The samples were covered and shaken at room temperature, 300 rpm for 2 h (Heidolph Tiramax 1000). After that, each sample was transferred into a filter plate (MSSBLPC, Multiscreen Filter plate, Merck kGaA) and filtered (Vaccum Manifold, Merck kGaA, Darmstadt, Germany). The filtrates were transferred into UV-Star microplates (Greiner bio-one) and acetonitrile (AcN) was added to each well to avoid precipitation. The final solvent ratio was AcN:PBS 70:30. UV-Vis spectra were collected for each replicate by a Multiskan Sky UV-plate reader (Thermo Scientific, Waltham, MA, USA) in a wavelength range of 250–700 nm, with 1 nm steps. After blank substraction, the kinetic solubility of the samples was determined based on absorption at λ_max_ using a 5-point calibration curve for each sample recorded in 70:30 AcN:PBS solvent.

#### Determination of LOD, LOQ

Limit of detection (LOD) and limit of quantification (LOQ) were calculated with Equations (1) and (2) respectively, based on UV-Vis spectral data:

(1) $$\mathrm{LOD}=\frac{3.3\times \sigma }{s}\;$$

(2) $$\mathrm{LOQ}=\frac{10\times \sigma }{s}\;$$

where the σ value in the equations stands for the standard deviation of the absorption base line at 700 nm (where no sample-specific absorption was observed). The s value is the slope of the calibration curve recorded for each sample based on their absorption at λ_max_ (Table 4.)

### 3.2. BBB-Specific Permeability Assay

In vitro, blood-brain barrier-specific (BBB) permeability studies were carried out using the PAMPA technique. First, solutions with the nominal concentration of 250 μM were prepared as described in Section 3.1. The solutions were shaken for 1 h at room temperature, at 300 rpm. For preparing the artificial BBB-specific membrane 16 mg BPLE and 8 mg cholesterol were dissolved in 600 μL dodecane. Each well of the donor plate (MultiscreenTM-IP, MAIPNTR10, pore size 0.45 mm, Merck kGaA) was coated with 5 μL lipid solution, and fitted into the acceptor plate containing 300 μL PBS (pH 7.4) with 2.5% DMSO, and 150–150 μL of the PBS solutions (made from the DMSO stock solutions) were put on the donor plate’s artificial membrane. The sandwich plate system was covered with a tissue of wet paper and a plastic lid to avoid evaporation of the solvent, and it was incubated at 37 °C for 4 h. In the end, the donor solutions were filtrated (Vacuum Manifold, Merck kGaA), and the initial 250 μM solutions (c_D_(0)), the donor (c_D_(t)) and acceptor solution (c_A_(t)) were analyzed by a Multiskan Sky UV-plate-reader (Thermo Scientific) in the wavelength range of 250–700 nm, with 1 nm steps.

BBB permeability was calculated using the effective permeability equation used for iso-pH conditions described by A. Avdeef [50]:

(3) $$P_{e}=\frac{-2.303}{A\cdot \left(t-\tau_{\mathrm{ss}}\right)}\cdot \left(\frac{1}{1+r_{v}}\right)\cdot \mathrm{lg}\left[-r_{v}+\left(\frac{1+r_{v}}{1-\mathrm{MR}}\right)\cdot \frac{c_{D}\left(t\right)}{c_{D}\left(0\right)}\right]$$

(4) $$\mathrm{MR}=1-\frac{c_{D}\left(t\right)}{c_{D}\left(0\right)}-\frac{V_{A}c_{A}\left(t\right)}{V_{D}c_{D}\left(0\right)}$$

where A is the filter area (0.3 cm^2^), V_D_ and V_A_ are the volumes in the donor (0.15 cm^3^) and acceptor phase (0.3 cm^3^), t is the incubation time (s), τ_SS_ is the time to reach steady-state (s), c_D_(t) is the concentration of the compound in the donor phase at time point t (mol/cm^3^), c_D_(0) is the concentration of the compound in the donor phase at time point zero (mol/cm^3^), c_A_(t) is the concentration of the compound in the acceptor phase at time point t (mol/cm^3^), $r_{\upsilon }$ is the aqueous compartment volume ratio (V_D_/V_A_).

### 3.3. Determination of Water Dispersibility

About 2 mg of the examined substance was dispersed in 40 mL of distilled water and the suspension was sonicated for 2 min. The addition of water was continued with 10 mL portions, after each portion the mixture was sonicated for 2 min. This process was continued till a homogenous solution was observed by naked eye. Then, the solution was let to stand for an hour to inspect any precipitate formation, but no changes occurred in any of the cases.

### 3.4. Determination of Hydrodynamic Diameter and Colloidal Stability

Size determination of aggregates and the colloidal stability of the sample dispersions were determined by dynamic light scattering measurements, where the hydrodynamic diameter of particles, and zeta potential values were collected. The number-weighted size distribution indicated one main peak in each case, the polydispersity index (PDI) was calculated as sd^2^/mean size^2^. For the particle size analysis Malvern Zetasizer Nano S instrument and for the zeta potential determination Malvern Zetasizer Nano Z instrument were used (Malvern Panalytical Ltd., Great Malvern, Worcestershire, UK). The measurements were carried out in autocorrelation mode, and the following parameters were kept constant: scattering angle 173°, attenuator 11 and its factor 0.0146, measurement position 4.65 mm. The concentrations of sample dispersions were between 0.3125 and 5 µM. The dispersion medium was PBS. Each samples was examined after 6 min incubation period at 37 °C.

### 3.5. Determination of Antioxidant Capacity

#### 3.5.1. TEAC Assay Using ABTS Radical

The TEAC values were determined in isotonic phosphate buffer of pH 7.4. The buffer solution was made by dissolving 9.55 g of DPBS powder in distilled water to form 1L of solution (composition: 0.138 M NaCl, 0.0027 M KCl, 0.0081 M Na_2_HPO_4_, 0.0015 M KH_2_PO_4_). The assay was performed according to a literature process with slight modifications [44,45]. The ABTS^•+^ radical cation was produced by reacting 7 mM 2,2′-azino-bis(3-ethylbenzothiazoline-6-sulfonic acid) and 2.45 mM potassium persulfate in water. The stock ABTS^•+^ solution was prepared 12–16 h before the experiments, and stored at room temperature in dark. The absorbance of the ABTS^•+^ solution was set to 0.70 ± 0.05 at 734 nm by a ca. 100-fold dilution with phosphate-buffered saline (PBS). Trolox and melatonin were dissolved in ethanol, the carotenoids and their conjugates in tetrahydrofuran (THF) or in dimethylsulfoxide (DMSO) to get 2.5 × 10^−4^ M stock solutions. The stock solutions were diluted with PBS such that equal amounts of the diluted solutions were added to the ABTS^•+^ reagent to obtain final antioxidants concentrations of 0.3125, 0.625, 1.25, 2.50, 3.75, 5 μM, respectively. In the blank (0 μM), the solutions of the antioxidants were substituted with THF or DMSO.

The antioxidants were incubated with ABTS^•+^ solution at 37 °C for 6 min. During the reaction of ABTS^•+^ with the antioxidants the absorbance of the solution decreases. The percentage inhibition of absorbance at 734 nm was calculated as (A_0_ − A_antioxidant_)/A_0_, where A_0_ is the absorbance of the ABTS^•+^ solution and A_antioxidant_ is the absorbance measured after the addition of the antioxidant. The determinations were carried out at each concentration in triplicate. The calculated values were plotted against the final concentration of the antioxidants. The slopes of the curves were compared with that for trolox, the TEAC value is the ratio of the slopes for the antioxidant and for trolox.

#### 3.5.2. FRAP Assay for the Antioxidant Capacity

A modified literature process was used [46]. The FRAP reagent should also be prepared freshly each day. 2,4,6-Tris(2-pyridyl)-*s*-triazin (TPTZ) was dissolved in a 200 mM HCl solution and diluted with water to get a final 40 mM HCl and 10 mM TPTZ concentration. For the preparation of the FRAP reagent 30 mL of 20 mM FeCl_3_ solution and 30 mL of 10 mM TPTZ solution were mixed, and 300 mL of 300 mM acetate buffer with pH 3.0 was added (a pH lower than usual was used to avoid the precipitation of the TPTZ). The FRAP reagent was incubated at 37 °C for 15 min. 150 μL of antioxidant dissolved in THF was mixed with 2.85 mL FRAP reagent solution, and incubated at 37 °C for 15 min in dark. Measurements were carried out at each antioxidant concentration (0, 7.5; 10; 12.5; 15; 17.5 μM final concentration) in triplicate. In the 0 μM solution the antioxidant solution was substituted with acetate buffer (pH 3.0). The FRAP was determined at 600 nm: the absorbance values were plotted as a function of concentration of the sample compounds and that of Trolox for the standard reference data. The FRAP values were calculated as in the ABTS assays, i.e., the slope of the concentration-absorbance lines for the carotenoid derivatives was divided by that for the trolox.

#### 3.5.3. Statistical Analysis

All experiments were done in triplicate. Data were expressed as means ± SD. For the comparison of the means one way ANOVA with Student-Newman-Keuld post-hoc test was calculated, and to analyze the homogeneity of variance Levene statistics was implemented by using SPSS 26.0 (SPSS, Chicago, IL, USA). A difference was considered statistically significant at *p* < 0.05.

## 4. Conclusions

Carotenoid succinates and their conjugates with melatonin were synthetized in an efficient way, to enhance the direct and indirect antioxidant effect of carotenoids. Nevertheless, the antioxidant capacity showed a solvent dependence in the ABTS assays. In a phosphate-buffered saline, which ensures an intracellular-like medium, both the hydrophilic succinates and the hydrophobic melatonin conjugates of lutein, capsanthin and 8′-apo-β-carotenol surpassed the TEAC values of the parent carotenoids, in the case of zeaxanthin and β-cryptoxanthin the conjugates gave similar or lower TEAC as the native carotenoids. According to the FRAP measurements at a lower pH, the best antioxidants were the bismelatonin derivatives of lutein and capsanthin. It is also worth mentioning the considerable differences in the antioxidant behaviour of lutein and zeaxanthin, and their conjugates. The aggregations in the aqueous media most probably influence the antioxidant capacities. To avoid aggregation and on the basis of the determined physicochemical properties, further investigation is needed to find a delivery system for the new conjugates.

The melatonin derivatives as hybrid/bifunctional molecules are expected to exert their full potential in oxidative stress protection in vivo, these assays including anti-inflammation tests on cell lines will be done in the near future. It remains to verify if melatonin-carotenoid conjugates can cross in vivo blood-brain barrier and blood-retina barrier, as well as other possible effects such as anti-inflammatory, immunomodulatory and oncostatic effects.

## Figures and Tables

**Table 1:** Preparation of Succinate Esters of Hydroxy Carotenoids.

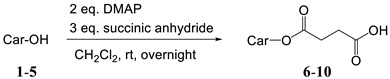	
Carotenoid (Car-OH)	Product (Yield)
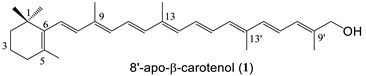	**6** succinate (92%) ^1^
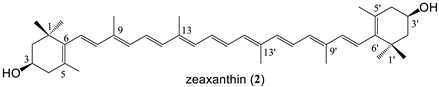	**7** bissuccinate (82%)
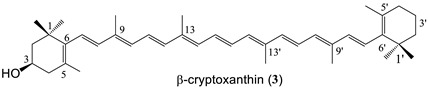	**8** succinate (78%)
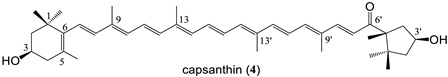	**9** bissuccinate (75%)
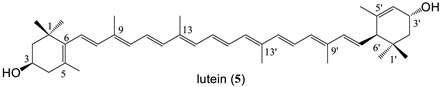	**10** bissuccinate (79%)

^1^ in two steps from 8′-apo-β-carotenal.

**Table 2:** Predicted (Using ACD/Labs Percepta Software [34]) and in Vitro Experimental Physicochemical and Early ADME Profile of Investigated Carotenol Derivatives.

	Predicted Parameters								Experimental Data	
Compound	Mw	Strongest pK_a,acid_^a^	logP/logD_7.4_^a^	HBD/ HBA	TPSA Å^2^	Aq.Sol. ^b^ mg/mL	Caco-2 Permeability Pe 10^−6^ cm/s [35]	logBB [36]	Kin.Sol. ^c^ μM	BBB-PAMPA Pe 10^−6^ cm/s
**1**	417	-	8.6	1/1	17.1	5 × 10^−5^	3.2	0.03	ND	ND
**2**	569	-	10.8	2/2	40.5	7 × 10^−5^	0.2	−0.96	ND	ND
**3**	553	-	12.1	1/1	20.2	3 × 10^−5^	0.1	−2	ND	ND
**4**	585	-	9.3	2/3	57.5	2 × 10^−5^	0.3	−0.58	ND	ND
**5**	569	-	10.4	2/2	40.5	1 × 10^−4^	0.2	−0.66	ND	ND
**6**	519	4.4	9.3/6.4	1/3	63.6	2 × 10^−2^	5.8	−1.08	52.3 ± 2.4	ND
**7**	769	4.2	11.4/6.7	2/7	127.2	4 × 10^−1^	2.2	−2	0.6 ± 0.1	ND
**8**	653	4.4	12.5/9.6	1/3	63.6	2 × 10^−3^	0.3	−2	1.3 ± 0.1	ND
**9**	785	4.4	10.1/5.3	2/8	144.3	5 × 10^−2^	6.7	−2	ND	ND
**10**	769	4.2	11.2/6.4	2/7	127.2	2 × 10^−1^	2.9	−2	ND	ND
**11**	669	4.4	11.0/8.1	2/4	83.8	4 × 10^−3^	0.4	−2	ND	ND
**13**	898	-	12.2	4/5	129.8	2 × 10^−7^	0.1	−2	ND	ND
**14**	1228	-	13.5	6/8	219.0	3 × 10^−10^	0.1	−2	ND	ND
**15**	882	-	13.7	3/4	109.5	6 × 10^−8^	0.1	−2	ND	ND
**16**	1228	-	13.1	6/8	219.0	4 × 10^−10^	0.1	−2	ND	ND
**17**	1244	-	11.8	6/9	231.1	8 × 10^−11^	0.1	−2	ND	ND
**18**	748	-	10.2	3/4	109.5	1 × 10^−5^	0.1	−1	ND	ND
Melatonin	232	-	1.7	2/2	54.1	9 × 10^−1^	58.2	−0.22	123.2 ± 9.4	2.97 ± 1.18

^a^ Calculated using logP/logD_7.4_ (Consensus and pK_a_ (Classic) settings within Percepta package. ^b^ aqueous solubility: logS_pH6.5_ (at intestinal conditions) using Drug Profiler unit of Percepta Package. ^c^ kinetic solubility values after 2 h, at 37 °C in PBS, pH 7.4.

**Table 3:** Water-Dispersibility of Carotenoid Succinates.

Succinate	Water Dispersibility (mg/100 mL)
8′-apo-β-carotenol succinate (**6**)	1.5
zeaxanthin bissuccinate (**7**)	1.3
β-cryptoxanthin succinate (**8**)	less than 0.5
capsanthin bissuccinate (**9**)	less than 0.6
lutein bissuccinate (**10**)	1.5
zeaxanthin monosuccinate (**11**)	less than 0.3
underivatized carotenoids (**1**–**5**)	0.05–0.1

**Table 4:** Analytical Data for Each Compounds: Wavelength of Maximum Absorbance (λ_max_), Slope and R^2^ of Fitted Calibration Curve, Calculated Limit of Detection (LOD) and Limit of Quantification (LOQ).

Compound	λ_max_ (nm)	Slope of Fitted Calibration Curve (s)	R^2^ of Fitted Calibration Curve	LOD (μM)	LOQ (μM)
**1**	467	0.0119	0.999	2.20	6.67
**2**	467	0.0009	0.995	29.08	88.14
**3**	457	0.0013	0.999	20.14	61.02
**4**	472	0.0256	0.991	1.02	3.10
**5**	452	0.0084	0.995	3.12	9.44
**6**	459	0.0252	0.998	1.04	3.15
**7**	456	0.0246	0.993	1.06	3.22
**8**	483	0.0190	0.985	1.38	4.17
**9**	459	0.0124	0.998	2.11	6.40
**10**	452	0.0347	0.999	0.75	2.29
**11**	434	0.0267	0.999	0.98	2.97
**12**	280	0.0021	0.999	12.46	37.77
**13**	458	0.0258	0.996	1.01	3.07
**14**	457	0.0380	0.999	0.69	2.09
**15**	452	0.0085	0.987	3.08	9.33
**16**	452	0.0282	0.999	0.93	2.81
**17**	478	0.0126	0.999	2.08	6.30
**18**	431	0.0015	0.998	17.45	52.88

## Data Availability

The data presented in this study are available in the Appendix A of this article and in the Supplementary Materials.

## Supplementary Materials

UV-Vis spectra of the conjugates in different solvents, the hydrodynamic diameters and zeta-potentials of the aggregates, the statistical evaluation of the antioxidant measurements and HPLC chromatograms of all new substances are available online at https://www.mdpi.com/article/10.3390/molecules27154822/s1. Figures S1–S12: UV-spectra; Figures S13–S23: HPLC chromatograms, Table S1: TEAC values and their standard deviations determined by ABTS method; Table S2: FRAP values and their standard deviations.

## Sample Availability

Samples of all the prepared compounds are available from the authors.

## Appendix A. Experimental Section

Melting points were measured on a Stuart SMP30 apparatus and are uncorrected. NMR spectra were recorded with a Bruker Avance III Ascend 500 spectrometer (500/125 MHz for ^1^H/^13^C) in CDCl_3_, except otherwise indicated. Chemical shifts are referenced to Me_4_Si (^1^H), or to the residual solvent signals (^13^C). Molar masses were obtained by an Autoflex II MALDI instrument (Bruker Daltonics). DHB was used for the ionization of the samples. Mass spectra were monitored in positive mode with pulsed ionisation (λ = 337 nm; nitrogen laser, maximum pulse rate: 50 Hz). Spectra were measured in reflectron mode using a delayed extraction of 120 nsec. Spectra were the sum of 1000 shots, external calibration has been implemented. Data processing was executed with Flex Analysis software packages (version: 2.4., Billerica, MA, USA). The elemental analysis measurements were performed on a Fisons EA 1110 CHNS apparatus. The UV-Vis spectrophotometric measurements were implemented on a Jasco spectrophotometer model V-550 UV/Vis.

TLC was performed on Kieselgel 60 F_254_ (Merck), and the plates were visualised under UV light. For column chromatography Kieselgel 60 (VWR, particle size 0.063–0.200 mm) was used.

*General procedure for the synthesis of carotenoid succinates:*

The hydroxy carotenoids were dissolved in dry dichloromethane (20 mg/mL). To the stirred solution succinic anhydride (2.5 eq) and DMAP (3 eq) were added (eq per hydroxyl group). The mixture was stirred overnight under nitrogen atmosphere, in darkness, at room temperature. On the next day, 5–10 mL of 5% citric acid solution was added and stirred for additional 15 min. 200 mL dichloromethane was added and the organic phase was washed by 2 × 100 mL of 5% citric acid and 50 mL of brine, dried with Na_2_SO_4_, and evaporated in vacuum. The products were crystallized from dichlormethane/hexane. The obtained crystals were filtered out, dried in vacuum and stored in closed ampoules under argon. Crude 8′-apo-β-carotenol was freshly prepared from 8′-apo-β-carotenal each time [49], in this case, the yield is calculated for the two reactions together.

*8′-Apo-β-carotenol succinate* (**6**) Yield: 92% orange crystals. mp: 123–124 °C. λ_max_ (in THF): 410, 433, 458 nm. MS (MALDI-TOF, neg. mode, without matrix) *m/z* = 519 (M^+^). ^1^H-NMR δ ppm: 1.02 (s, 6H, H-16, H-17); 1.50 (m, 2H, H-2); 1.62 (m, 2H, H-3); 1.76 (s, 3H, H-18); 2.04 (m, 13H, H-4, H-19, H-19′, H-20, H-20′); 2.65 (m, 4H, 2CH_2_-succinate); 4.59 (s, 2H, H-8′); 6.15–6.63 (2m, 12H, olefinic). ^13^C-NMR δ ppm: 12.8 (C-19, C-20, C-19′); 14.7 (C-20′); 19.3 (C-3); 21.8 (C-18); 28.8–28.9 (2CH_2_-succinate, C-16, C-17); 33.1 (C-4); 34.3 (C-1); 39.6 (C-2); 70.38 (C-8′); 123.3–131.6, 132.2, 133.0, 135.7–138.6 (olefinic); 172.0 (C=O); 177.2 (COOH). Analysis calc. for C_34_H_46_O_4_: C 78.72, H 8.94; found C 78.10, H 9.11.

*Zeaxanthin bissuccinate* (**7**) Yield: 70%, orange crystals. mp: 152–153 °C. λ_max_ (in THF): 434, 458, 486 nm. MS (MALDI-TOF, neg. mode, without matrix) *m/z* = 769.3 (M^+^). ^1^H-NMR δ ppm: 1.05 (2s, 12H, H-17, H-17′, H-18, H-18′); 1.58 (t, 2H, H-2b, H-2′b); 1.72 (s, 6H, H-16, H-16′); 1.75 (t, 2H, H-2a, H-2′a); 1.96 (d, 12H, H-19, H-19′, H-20, H-20′); 2.13 (m, 2H, H-4b, H-4′b); 2.43 (m, 2H, H-4a, H-4′a) 2.66 (2m, 8H, 4CH_2_-succinate); 5.08 (m, 2H, H-3, H-3′); 6.1–6.65 (3m, 14H, olefinic). ^13^C-NMR δ ppm: 12.7–12.8 (C-19, C-19′, C-20, C-20′); 21.5 (C-18, C-18′); 28.5–28.9, 29.2, 30.0 (C-16, C-16′, C-17, C-17′, 4CH_2_-succinate); 36.7 (C-1); 38.3 (C-4); 43.9 (C-2); 69.1 (C-3, C-3′); 124.9–125.5, 130.1, 131.5, 132.6, 135.5, 136.4, 137.6, 137.9, 138.7 (olefinic); 171.8 (2C=O); 177.4 (2COOH). Analysis calc. for C_48_H_64_O_8_ C 74.97, H 8.39; found C 74.52, H 8.48.

*β-Cryptoxanthin succinate* (**8**) Yield: 78%, red crystals, mp: 142–143 °C. λ_max_ (in THF): 459, 486 nm. MS (MALDI-TOF, neg. mode, without matrix) *m/z* = 653 (M^+^). ^1^H-NMR δ ppm: 0.85–2.32 (m, 40H, H-16, H-16′, H-17, H-17′,H-2, H-2′, H-3′, H-18, H-18′, H-4′, H-4, H-19, H-20, H-19′, H-20′); 2.63 (m, 4H, 2CH_2_, succinate); 5.08 (m, 1H, H-3); 6.13–6.84 (m, 14H, olefinic). ^13^C-NMR δ ppm: 12.7–12.8 (C-19, C-19′, C-20, C-20′); 19.3 (C-3′); 21.4, 21.7 (C-18, C-18′); 28.5, 29.0, 30.0 (C-16, C-16′, C-17, C-17′); 28.9, 29.3 (2CH_2_-succinate); 33.1 (C-4′); 34.3, 36.7 (C-1, C-1′); 38.4 (C-4); 39.8 (C-2′); 44.0 (C-2); 69.1 (C-3); 124.9, 125.1, 125.2, 126.7 (C-7, C-7′, C-11, C-11′); 130.0, 130.2, 130.8, 131.5, 132.4, 132.7 (C-10, C-10′, C-14, C-14′, C-15, C-15′); 125.5, 129.4, 135.5, 136.1, 136.3, 136.6, 138.0 (C-5, C-5′, C-6, C-6′, C-9, C-9′, C-13, C-13′); 137.2, 137.7, 137.8, 138.7 (C-8, C-8′, C-12, C-12′); 171.8 (C=O); 177.1 (COOH). Analysis calc. for C_44_H_60_O_4_: C 80.94, H 9.26; found C 80.49, H 9.50.

*Capsanthin bissuccinate* (**9**) Yield: 75%, red crystals. mp: 143–144 °C. λ_max_ (in THF): 477 nm. MS (MALDI-TOF, neg. mode, without matrix) *m/z* = 785 (M^+^). ^1^H-NMR δ ppm: 0.87, 1.07, 1.10, 1.19 (4*s*, 12H, H-16, H-16′, H-17, H-17′), 1.32 (s, 3H, 18′-Me), 1.55–1.62 (m, 2H, H-2_ax_, H-4′β), 1.72 (*s*, 3H, H-18), 1.73–1.80 (m, 2H, H-2_eq_, H-2′β), 1.94, 1.96, 1.98 (3*s*, 12H, H-19, H-19′, H-20, H-20′), 2.06–2.16 (m, 2H, H-4_ax_, H-2′α), 2.44 (dd, 1H, *J* = 5.5 Hz, *J* = 17.4 Hz, H-4_eq_), 2.61–2.70 (m, 8H, 4CH_2_ succinate), 3.0 (dd, 1H, *J* = 8.8 Hz, *J* = 14.9 Hz, H-4′α), 5.08–5.13 (m, 1H, H-3), 5.28–5.32 (m, 1H, H-3′), 6.08–6.12 (m, 2H, H-7, H-8), 6.14 (d, 1H, *J* = 11.2 Hz, H-10), 6.27 (d, 1H, *J* = 11.2 Hz, H-14), 6.35–6.38 (m, 2H, H-12, H-14′), 6.42 (d, 1H, *J* = 15.0 Hz, H-7′), 6.50–6.73 (m, 6H, olefinic), 7.35 (d, 1H, *J* = 15.0 Hz, H-8′). ^13^C-NMR δ ppm: 12.7, 12.8, 20.8, 21.5, 24.8, 25.6, 28.5, 30.0, 32.5 (CH_2_-succinate); 36.7, 38.4, 42.3, 43.8, 44.1, 47.7, 70.0, 74.0, 120.6, 124.1, 125.5, 125.6, 128.0, 131.4, 131.7, 132.4, 133.59, 135.4, 136.0, 137.6, 137.5, 138.7, 141.0, 142.0, 147.2, 171.5 (m; 2*C*=O); 177.5 (2COOH). Analysis calc. for C_48_H_64_O_9_: C 73.44, H 8.22; found C 73.24, H 8.28.

*Lutein bissuccinate* (**10**) Yield: 70%, orange crystals. mp: 148–149 °C. λ_max_ (in THF): 429, 452, 481 nm. MS (MALDI-TOF, neg. mode, without matrix) *m/z* = 769 (M^+^). ^1^H-NMR δ ppm: 1.07 (m, 12H, H-16, H-16′, H-17, H-17′); 1.45–2.26 (m, 24H, H-2b, H-2′b, H-2a, H-2′a, H-4b, H-18, H-18′, H-19, H-19′, H-20, H-20′); 2.45 (m, 2H, H-4a, H-4a’); 2.67 (m, 8H, 4CH_2_-succinate); 5.2-5.4 (m, 2H, H-3, H-3′); 6.1-6.8 (2m, 14H, olefinic). ^13^C-NMR δ(ppm): 12.9–13.1 (C-19, C-19′, C-20, C-20′); 21.4, 22.9, 25.6 (C-18, C-18′); 28.5- 29.9; 33.3 (C-16, C-16′, C-17, C17′, 4CH_2_-succinate); 36.7, 38.3 (C-1, C1′); 39.1, 43.9, 54.9 (C-4, C-4′); 69.1–69.4 (C-3, C-3′); 119.6–140.8 (olefinic); 171.7–171.8 (2C=O); 177.8 (2COOH). Analysis calc. for C_48_H_64_O_8_ C 74.97, H 8.39, found C 74.66, H 8.48.

*Zeaxanthin monossuccinate* (**11**) Yield: 21%, orange crystals. mp: 137–138 °C. λ_max_ (in THF): 433, 458, 486 nm. MS (MALDI-TOF, neg. mode, without matrix) *m/z* = 669 (M^+^). ^1^H-NMR δ ppm: 1.10 (2*s*, 12H, H-17, H-17′, H-18, H-18′); 1.46 and 1.60 (2m, 2H, H-2b, H-2′b); 1.76 (s, 6H, H-16, H-16′); 1.78 and 1.85 (2m, 2H, H-2a, H-2′a); 1.99 (2s, 12H, H-19, H-19′, H-20, H-20′); 2.05 and 2.17 (2m, 2H, H-4b, H-4′b); 2.35 and 2.42 (2m, 2H, H-4a, H-4′a) 2.48 and 2.58 (2m, 4H, 2CH_2_-succinate); 3.93 (m, 1H, H-3′) 5.07 (m, 1H, H-3, H-3′); 6.12–6.75 (3m, 14H, olefinic). Analysis calc. for C_44_H_60_O_5_ C 79.00, H 9.04, found C 78.66, H 9.21.

**General procedure for the coupling with melatonin:**

The following equivalents correspond to one carboxyl group, for bissuccinates double amount was used from all reagents. A mixture of *N*-hydroxy-succinimide (0.2 eq) and the carotenoid succinate (1 eq.) was stirred in dry DMF (30 mg succinate/mL) for 10 min when DIPEA (5 eq.) and the amino-melatonin ∙ HCl (1.25 eq) were added. After another 10 min EDC ∙ HCl (1.5 eq) was added and the mixture was stirred overnight. The mixture was diluted with dichloromethane and was washed with brine three times, dried and evaporated. The product was purified on a silica gel column starting with a hexane:acetone 3:7 eluent mixture, in which methanol content was gradually increased from 1% to 10%. Products were dissolved in acetone and toluene, and were precipitated with hexane. For preparative TLC (Merck) the same solvent mixtures were used as for the columns.

*Zeaxanthin-monomelatonin conjugate* (**13**) Yield: 73% orange crystals. mp: 156–157 °C. λ_max_ (in THF): 432, 458, 486 nm. MS (MALDI-TOF, neg. mode, without matrix) *m/z* = 898 (M^+^). ^1^H-NMR δ ppm: 1.08 (2s, 12H, H-16, H-16′, H-17, H-17′); 1.60 (t, 2H, H-2b, H-2′b); 1.72, 1.79 (2s, 6H, H-18, H-18′); 1.80 (t, 2H, H-2a, H-2′a); 1.99 (m, 12H, H-19, H-19′, H-20, H-20′); 2.10 (m, 1H, H-4b or H-4′b); 2.42 (m, 3H, H-4a, H-4′a, H-4b or H-4′b); 2.47 and 2.68 (2m, 4H, 2CH_2_-succinate); 2.98 (m, 2H, H-11″); 3.61 (m, 2H, H-12″); 3.91 (m, 5H, H-10″, H-13″); 4.0 (m, 1H, H-3′); 5.05 (m, 1H, H-3); 6.1–6.65 (3m, 14H, olefinic); 6.88 (m, 1H, H-6″); 7.05 (m, 2H, H-4″, H-7″); 7.29 (m, 1H, H-2″); 8.05 (s, 2H, 2×NH). ^13^C-NMR δ ppm: 12.7–12.8 (C-19, C-19′, C-20, C-20′); 21.5, 21.6 (C-18, C-18′); 25.1 (C-11′); 28.5, 28.7, 29.7, 30.0 (C-16, C-16′, C-17, C-17′); 29.8, 30.9 (2CH_2_-succinate); 36.7 (C-4); 38.4 (C-1); 39.7, 43.4 (C-12″, C-13″); 44.0 (C-2); 56.0 (C-10″); 65.1 (C-3′); 69.2 (C-3); 100.7 (C-4″); 111.9, 112.3 (C-6″, C-7″); 112.5 (C-3″); 123.0 (C-2″); 126.2 (C-3a″); 127.8 (C-7a″); 124.8, 124.9, 125.6, 130.1, 131.3, 131.5, 132.6, 132.7, 135.5, 136.6, 137.7, 137.9, 138.8 (olefinic); 154.1 (C-5″); 168.6, 172.0 (2C=O, amides); 172.9 (C=O, ester). Analysis calc. for C_57_H_75_N_3_O_6_ C 76.22, H 8.42, N 4.68; found C 76.45, H 8.49, N 4.61.

*Zeaxanthin-bismelatonin conjugate* (**14**) Yield: 72% orange crystals. mp: 149–150 °C. λ_max_ (in THF): 433, 458, 486 nm. MS (MALDI-TOF, neg. mode, without matrix) *m/z* = 1227 (M^+^). ^1^H-NMR δ ppm: 1.05, 1.06 (2s, 12H, H-16, H-16′, H-17, H-17′); 1.56 (t, *J* = 12 Hz, 2H, H-2b, H-2′b); 1.69 (s, 6H, H-18, H-18′); 1.75–1.78 (m, 2H, H-2a, H-2′a); 1.96, 1.97 (2s, 12H, H-19, H-19′, H-20, H-20′); 2.13 (dd, *J* = 9.3 Hz, *J* = 17.0 Hz, 2H, H-4b, H-4′b); 2.39 (dd, *J* = 5.7 Hz, *J* = 17.0 Hz, 2H, H-4a, H-4′a); 2.43 (t, *J* = 6.6 Hz, 2CH_2_-succinate); 2.65–2.68 (m, 4H, 2CH_2_-succinate); 2.95 (t, *J* = 6.8 Hz, 4H, 2 H-11″); 3.55–3.61 (m, 4H, 2 H-12″); 3.83–3.89 (m, 10H, 2CH_3_-10″, 2 H-13″); 5.00–5.06 (m, 2H, H-3, H-3′); 6.04–6.12 (m, 4H, H-7, H-7′, H-8, H-8′); 6.15 (d, *J* = 11.4 Hz, 2H, H-10, H-10′); 6.26 (d, *J* = 9.4 Hz, 2H, H-14, H-14′); 6.35–6.38 (m, 2H, H-12, H-12′); 6.61–6.66 (m, 8H, H-11, H-11′, H-15, H-15′); 6.86 (dd, *J* = 2.4 Hz, *J* = 8.8 Hz, 2H, H-6″); 7.02 (dd, *J* = 2.3 Hz, *J* = 11.1 Hz, 4H, H-4″, H-2″); 7.26 (d, J = 8.7 Hz, 2H, H-7″); 8.02 (s, 2H, NH). ^13^C-NMR δ ppm: 12.7, 12.8 (C-19, C-19′, C-20, C-20′); 21.5 (C-18, C-18′); 25.1 (2C-11″); 28.5, 30.0 (C-16, C-16′, C-17, C-17′); 29.7, 30.8 (4CH_2_-succinate); 36.7 (C-1, C-1′); 38.4 (C-4, C-4′); 43.4 (2C-12″); 44.0 (C-2, C-2′); 56.0 (2C-10″); 69.2 (C-3, C-3′); 100.7 (2C-4″); 112.0, 112.3 (2C-6″, 2C-7″); 112.6 (2C-3″); 123.0 (2C-2″); 125.0, 125.1 (C-7, C-7′, C-11, C-11′); 125.4 (C-5, C-5′); 127.8 (2C-3a″, 2C-7a″); 130.1, 131.5, 132.7 (C-10, C-10′, C-14, C-14′, C-15, C-15′), 135.5, 136.5 (C-9, C-9′, C-13, C-13′); 137.9 (C-6, C-6′); 137.8, 138.7 (C-8, C-8′, C-12, C-12′); 154.1 (C-5″); 168.7, 171.9 (4C=O, amides); 172.9 (2C=O, esters). Analysis calc. for C_74_H_94_N_6_O_10_ C 72.40, H 7.72, N 6.85; found C 72.62, H 7.67, N 6.89

*Cryptoxanthin-melatonin conjugate* (**15**) Yield: 55%, red crystals. mp: 153–154 °C. λ_max_ (in THF): 435, 457, 482 nm. MS (MALDI-TOF, neg. mode, without matrix) *m/z* = 882 (M^+^). ^1^H-NMR δ ppm: 1.09 (2s, 12H, H-16, H-16′, H-17, H-17′); 1.60 (m, 4H, H-3, H-2a, H-2b, H-2′b); 1.72, 1.79 (2s, 6H, H-18, H-18′); 2.04 (m, 15H, H-2′a, H-4, H-19, H-19′, H-20, H-20′); 2.15 (m, 1H, H-4′a); 2.41 (m, 1H, H-4′b); 2.47 and 2.70 (2m, 4H, 2CH_2_-succinate); 2.98 (m, 2H, H-11″); 3.62 (m, 2H, H-12″); 3.92 (m, 5H, H-10″, H-13″); 5.05 (m, 1H, H-3′); 6.05–6.67 (3m, 14H, olefinic); 6.90 (m, 1H, H-6″); 7.06 (m, 2H, H-4″, H-7″); 7.30 (m, 1H, H-2″); 8.01 (s, 1H, NH). ^13^C-NMR δ ppm: 12.7-12.8 (C-19, C-19′, C-20, C-20′); 19.3 (C-3); 21.4, 21.7 (C-18, C-18′); 25.1 (C-11′); 28.5, 29.0, 30.0 (C-16, C-16′, C-17, C-17′); 29.8, 30.9 (2CH_2_-succinate); 33.2 (C-4); 34.2 (C-1); 36.7 (C-4′); 38.4 (C-1′); 39.8, 43.4 (C-2, C-12″, C-13″); 44.0 (C-2′); 56.0 (C-10″); 69.1 (C-3′); 100.8 (C-4″); 111.9, 112.3 (C-6″, C-7″); 112.5 (C-3″); 123.0 (C-2″); 126.2 (C-3a″); 127.8 (C-7a″); 124.8, 124.9, 125.6, 130.1, 131.3, 131.5, 132.6, 132.7, 135.5, 136.6, 137.7, 137.9, 138.8 (olefinic); 154.1 (C-5″); 168.7, 171.9 (2C=O, amides); 172.9 (C=O, ester). Analysis calc. for C_57_H_75_N_3_O_5_ C 77.60, H 8.57, N 4.76; found C 77.70, H 8.47, N 4.61.

*Lutein-bismelatonin conjugate* (**16**) Yield: 48% orange crystals. mp: 123–124 °C. λ_max_ (in THF): 428, 453, 482 nm. MS (MALDI-TOF, neg. mode, without matrix) *m/z* = 1227 (M^+^). ^1^H-NMR δ ppm: 1.08 (m, 12H, H-16, H-16′, H-17, H-17′); 1.45–2.0 (2m, 24H, H-2b, H-2′b, H-2a, H-2′a, H-4b, H-18, H-18′, H-19, H-19′, H-20, H-20′); 2.38 (m, 1H, H-4a); 2.43 (m, 1H, H-6′); 2.47 and 2.67 (2m, 8H, 4CH_2_-succinate); 2.98 (m, 4H, H-11″); 3.61 (m, 4H, H-12″); 3.91 (m, 10H, H-10″, H-13″); 5.2-5.4 (m, 2H, H-3, H-3′); 6.1-6.65 (3m, 14H, olefinic); 6.95 (m, 2H, H-6″); 7.05 (m, 4H, H-4″, H-7″); 7.27 (m, 2H, H-2″); 8.04 (s, 2H, NH). ^13^C-NMR δ ppm: 12.7–12.9 (C-19, C-19′, C-20, C-20′); 21.5 (C-18, C-18′); 25.1 (C-11″); 28.5, 30.0 (C-16, C-16′, C-17, C-17′); 29.8, 30.8 (4CH_2_-succinate); 36.7 (C-4); 38.3 (C-1); 39.7, 43.4 (C-12″, C-13″); 44.0 (C-2, C-2′); 53.8 (C-10″) 56.0 (C-6′); 69.2, 69.6 (C-3, C-3′); 100.7 (C-4″); 112.0, 112.3 (C-6″, C-7″); 112.6 (C-3″); 123.0 (C-2″); 125.4 (C-3a″); 128.3 (C-7a″); 114.0, 124.9, 125.6, 130.1, 131.5, 132.7, 135.5, 136.5, 137.7, 137.9, 138.7 (olefinic); 154.0 (C-5″); 168.8, 172.0 (4C=O, amides); 173.0 (2C=O, esters) Analysis calc. for C_74_H_94_N_6_O_10_ C 72.40, H 7.72, N 6.85; found C 72.29, H 7.64, N 6.93.

*Capsanthin-dimelatonin conjugate* (**17**) Yield: 57% red crystals. mp: 130–131 °C. λ_max_ (in THF): 476 nm. MS (MALDI-TOF, neg. mode, without matrix) *m/z* = 1243 (M^+^). ^1^H-NMR δ ppm: 0.87, 1.08, 1.09, 1.18 (4s, 12H, H-16, H-16′, H-17, H-17′), 1.33 (s, 3H, 18′-Me), 1.47 (m, 2H, H-2a, H-4′b), 1.72 (s, 3H, H-18), 1.73–1.80 (m, 2H, H-2b, H-2′a), 1.97, 1.99, 2.0, 2.01 (4s, 12H, H-19, H-19′, H-20, H-20′), 2.06–2.18 (m, 2H, H-4a, H-2′b), 2.42 (m, 1H, H-4_eq_), 2.45, 2.67 (2m, 8H, 4CH_2_-succinate), 2.98 (m, 3H, H-4′b, H-11″), 3.62 (m, 2H, H-12″); 3.91 (m, 5H, H-10″, H-13″); 5.05 (m, 1H, H-3), 5.24 (m, 1H, H-3′), 6.1–6.75 (3m, 13H, olefinic) 6.90 (m, 1H, H-6″); 7.06 (m, 2H, H-4″, H-7″); 7.29 (m, 1H, H-2″), 7.35 (m, 1H, H-8′). 8.02, 8.20 (2s, 2H, NH). ^13^C-NMR δ ppm: 12.7; 12.8; 20.6; 21.4; 24.8; 25.1; 25.5; 28.53; 29.7; 30.9; 30.0, 31.6 (4CH_2_-succinate); 38.4; 39.7, 43.4 (C-12″; C-13″) 42.3; 43.7; 44.0; 47.5; 56.0 (C-10″); 69.0; 74.0; 100.9 (C-4″); 112.0, 112.2 (C-6″, C-7″); 112.3 (C-3″); 120.6; 123.0 (C-2″); 124.0; 125.5 (C-3a″); 128.3 (C-7a″); 128.0; 131.4; 131.7; 132.4; 133.59; 135.4; 136.0; 137.6; 137.5; 138.7; 141.0; 142.0; 147.2; 154.1 (C-5″); 168.7, 172.0 (4C=O, amides); 172.9 (2C=O, esters). Analysis calc. for C_74_H_94_O_6_N_11_ C 71.47, H 7.62, N 6.76; found C 71.67, H 7.51, N 6.70.

*8′-Apo-**β**-carotenol-melatonin conjugate* (**18**) Yield: 51 % orange crystals. mp: 123–124 °C. λ_max_ (in THF): 407, 432, 457 nm. MS (MALDI-TOF, neg. mode, without matrix) *m/z* = 748 (M^+^). ^1^H-NMR δ ppm: 1.03 (s, 6H, H-16, H-17); 1.49 (m, 2H, H-2); 1.58 (s, 12H, H-19, H-19′, H-20, H-20′); 1.63 (m, 2H, H-3); 1.72 (s, 3H, H-18); 1.81 (s, 3H, H-20′), 1.93, 1.98 (2s, 9H, H-19, H-19′, H-20); 2.02 (t, 2H, H-4); 2.49, 2.73 (m, 4H, 2CH_2_-succinate); 2.98 (m, 2H, H-11″); 3.61 (m, 2H, H-12″); 3.92 (m, 5H, H-10″, H-13″); 4.54 (s, 2H, H-8′); 6.15–6.70 (2m, 12H, olefinic); 6.90 (m, 1H, H-6″); 7.06 (m, 2H, H-4″, H-7″); 7.30 (m, 1H, H-2″); 8.01 (s, 1H, NH). ^13^C-NMR δ ppm: 12.8 (C-19, C-20, C-19′, C-20′); 19.3 (C-3); 21.8 (C-18); 28.8-28.9 (2CH_2_-succinate, C-16, C-17); 33.2 (C-4); 34.3 (C-1); 39.8, 43.4 (C-2, C-12″, C-13″); 56.0 (C-10″); 70.9 (C-8′); 100.8 (C-4″); 111.9, 112.3 (C-6″, C-7″); 112.5 (C-3″); 123.0 (C-2″); 126.3 (C-3a″); 127.8 (C-7a″); 128.0–138.6 (m, olefinic); 154.1 (C-5″); 168.6, 171.9 (2C=O, amides); 172.9 (C=O, ester). Analysis calc. for C_47_H_61_N_3_O_5_ C 75.47, H 8.22, N 5.62; found C 75.70, H 8.38, N 5.51.
