# Supplementary material for: Synthesis, Pharmacokinetic Characterization and Antioxidant Capacity of Carotenoid Succinates and Their Melatonin Conjugates
Source: Molecules. 2022 Jul 28;27(15):4822. doi: 10.3390/molecules27154822 (PMC9369794; doi:10.3390/molecules27154822)
Supplement: Supplementary file 1 [file molecules-27-04822-s001.zip › molecules-1823944-supplementary.pdf]

Supplementary materials for the article

Synthesis, Pharmacokinetic Characterization and Antioxidant Capacity of Carotenoid Succinates and Their Melatonin Conjugates

Dalma Czett [1], Katalin Böddi [1], Veronika Nagy [1], Anikó Takátsy [1], József Deli [1,2], Paul Tone [3], György T. Balogh [4,5], Anna Vincze [4] and Attila Agócs [1,*]

[1] Department of Biochemistry and Medical Chemistry, Medical School, University of Pécs, Szigeti út 12, H-7624 Pécs, Hungary; czett.dalma@gmail.com (D. C.); katalin.boddi@aok.pte.hu (K.B.); vera.nagy@aok.pte.hu (V.N.); aniko.takatsy@aok.pte.hu (A.T.); jozsef.deli@aok.pte.hu (J.D.)

[2] Department of Pharmacognosy, Faculty of Pharmacy, University of Pécs, Rókus u. 2, H-7624 Pécs, Hungary

[3] Department of Medicine, Richmond University Medical Center, Staten Island, NY 10310, USA; paultonemd@gmail.com

[4] Department of Chemical and Environmental Process Engineering, Budapest University of Technology and Economics, Műegyetem rkp. 3, H-1111 Budapest, Hungary; balogh.gyorgy@vbk.bme.hu (G.T.B.); vincze.anna@edu.bme.hu (A.V.)

[5] Institute of Pharmacodynamics and Biopharmacy, Faculty of Pharmacy, University of Szeged, Eötvös u. 6, H-6720 Szeged, Hungary

* Correspondence: attila.agocs@aok.pte.hu

1. Study of aggregation by UV-Vis spectrophotometry 2

2. Dinamic light scattering 6

3. Statistical analysis of the antioxidant measurements by ABTS method 9

4. Statistical analysis of the antioxidant measurements by FRAP method 10

5. ^{1}H and ^{13}C-dept NMR spectra of the bismelatonin conjugate of zeaxanthin (14) 12

6. HPLC chromatograms of the synthesized compounds 14

1. Study of Aggregation by UV-Vis Spectrophotometry

Starting from THF solutions: each conjugate was dissolved in freshly distilled THF and the solutions were diluted either with THF or THF-PBS mixtures to obain final solutions of identical carotenoid concentration.

Starting from DMSO solutions: each conjugate was dissolved in DMSO to obtain $2.5 \cdot 10^{-4}$ M stock solutions. These stock solutions were added to PBS to give final carotenoid concentrations of 0.3125, 0.625, 1.25, 2.50, 3.75 and 5 μM, respectively.

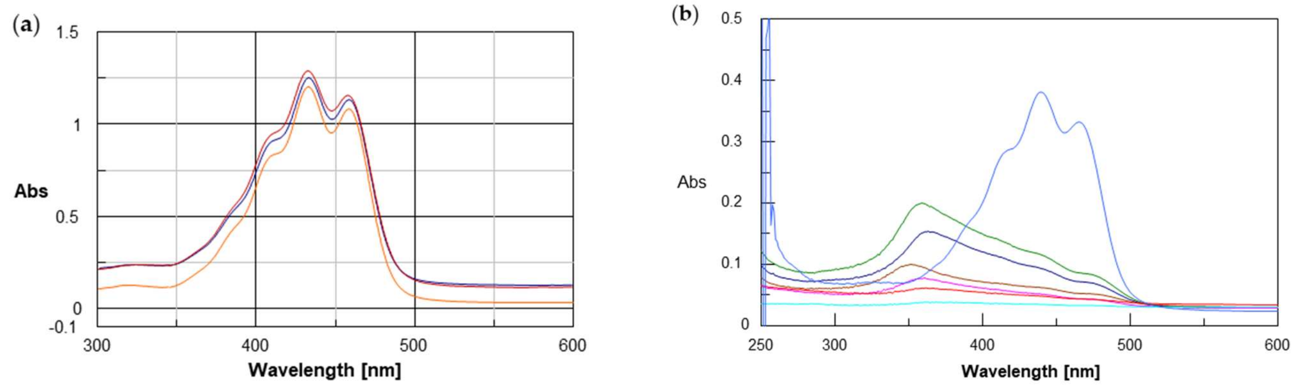

Figure S1. UV-spectra of 8′-apo-β-carotenol succinate (**6**) (**a**) in THF and in THF-PBS mixtures (1:1, 1:3); (**b**) in DMSO and in DMSO-PBS mixtures (5 μM, 3.75 μM, 2.5 μM, 1.25 μM, 0.625 μM and 0.3125 μM).

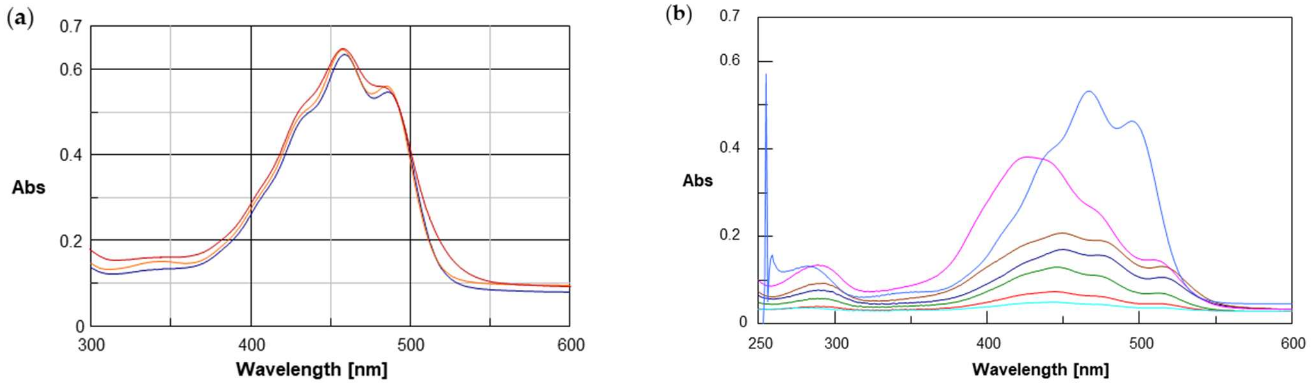

Figure S2. UV-spectra of zeaxanthin bissuccinate (**7**) (**a**) in THF and in THF-PBS mixtures (1:1, 1:3); (**b**) in DMSO and in DMSO-PBS mixtures (5 μM, 3.75 μM, 2.5 μM, 1.25 μM, 0.625 μM and 0.3125 μM).

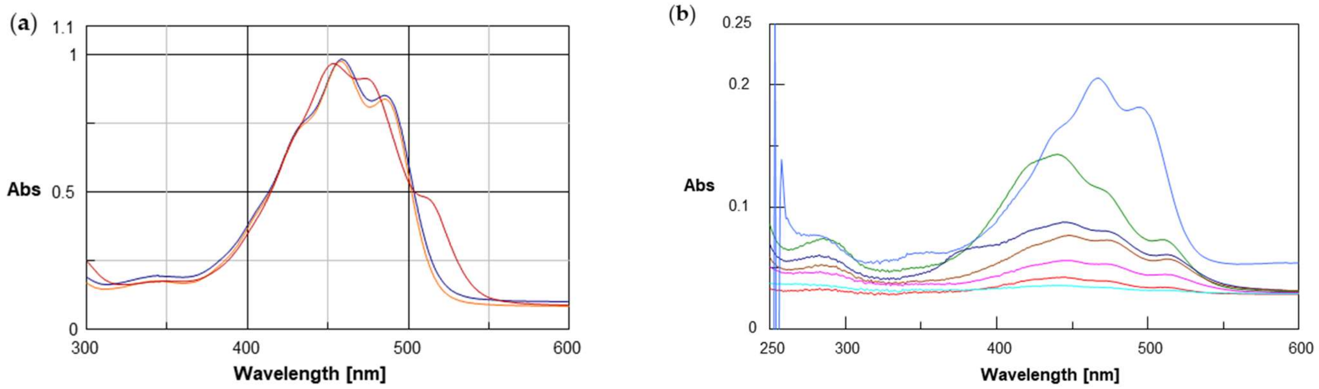

Figure S3. UV-spectra of zeaxanthin monosuccinate (**11**) (**a**) in THF and in THF-PBS mixtures (1:1, 1:3); (**b**) in DMSO and in DMSO-PBS mixtures (5 μM, 3.75 μM, 2.5 μM, 1.25 μM, 0.625 μM and 0.3125 μM).

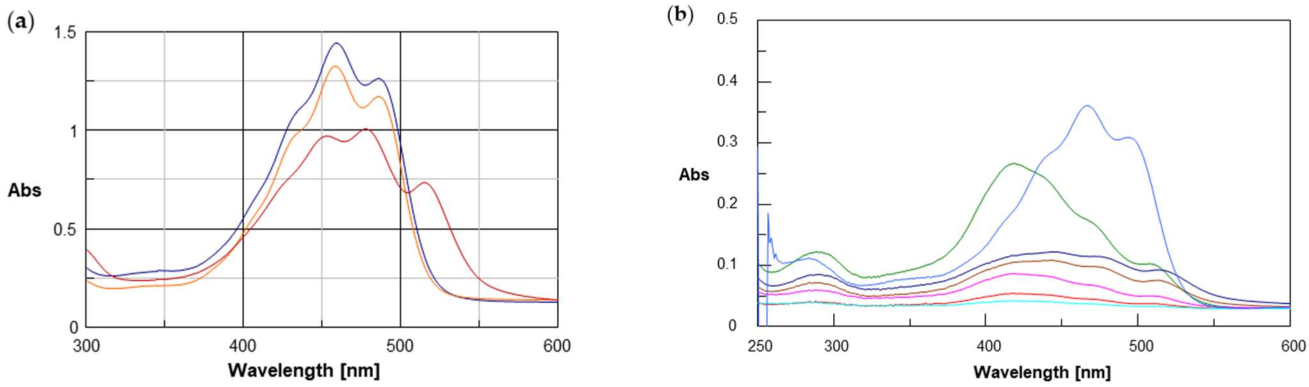

Figure S4. UV-spectra of β-cryptoxanthin succinate (**8**) (**a**) in THF and in THF-PBS mixtures (1:1, 1:3); (**b**) in DMSO and in DMSO-PBS mixtures (5 µM, 3.75 µM, 2.5 µM, 1.25 µM, 0.625 µM and 0.3125 µM).

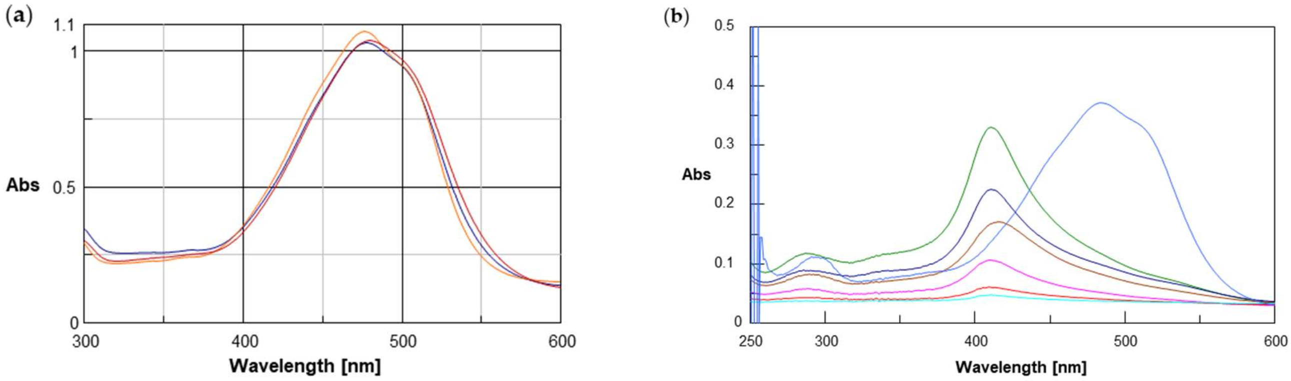

Figure S5. UV-spectra of capsanthin bissuccinate (**9**) (**a**) in THF and in THF-PBS mixtures (1:1, 1:3); (**b**) in DMSO and in DMSO-PBS mixtures (5 µM, 3.75 µM, 2.5 µM, 1.25 µM, 0.625 µM and 0.3125 µM).

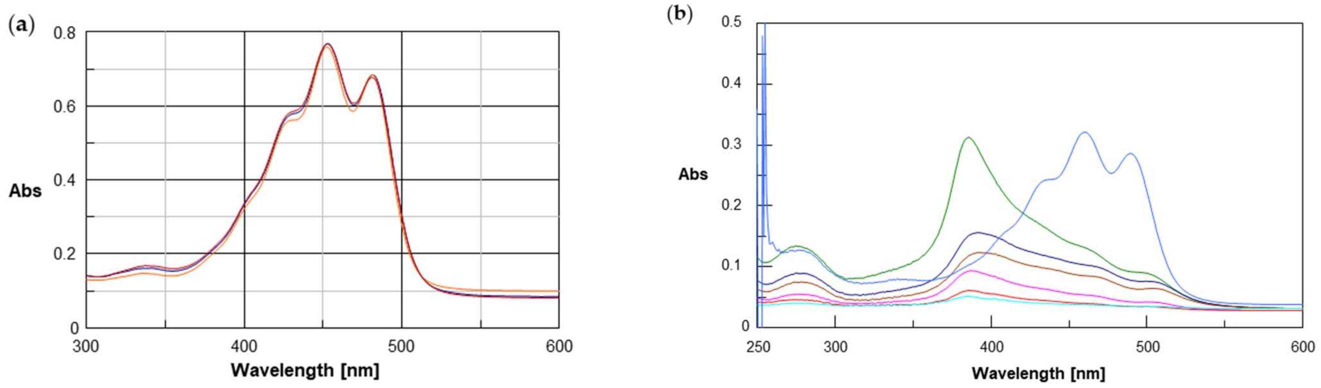

Figure S6. UV-spectra of lutein bissuccinate (**10**) (**a**) in THF and in THF-PBS mixtures (1:1, 1:3); (**b**) in DMSO and in DMSO-PBS mixtures (5 µM, 3.75 µM, 2.5 µM, 1.25 µM, 0.625 µM and 0.3125 µM).

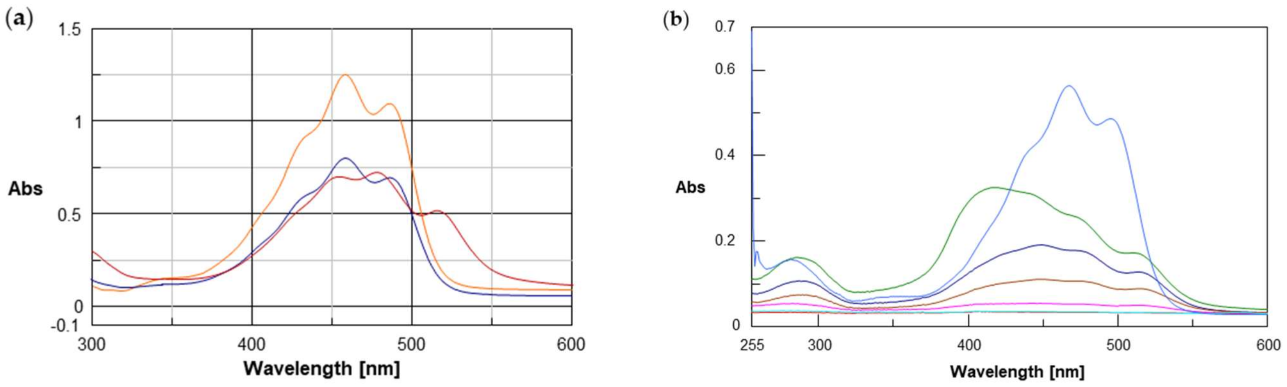

Figure S7. UV-spectra of zeaxanthin–monomelatonin conjugate (**13**) (**a**) in THF and in THF-PBS mixtures (1:1, 1:3); (**b**) in DMSO and in DMSO-PBS mixtures (5 µM, 3.75 µM, 2.5 µM, 1.25 µM, 0.625 µM and 0.3125 µM).

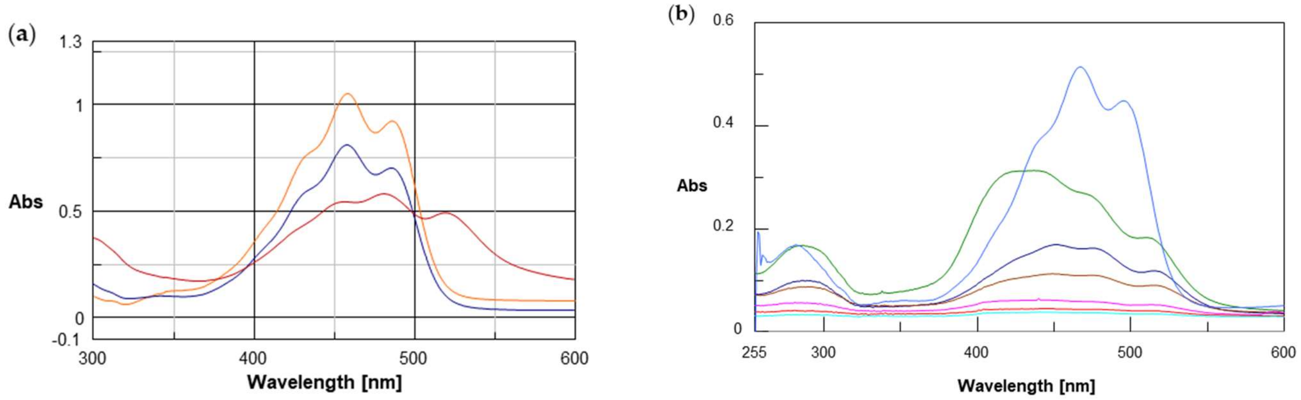

Figure S8. UV-spectra of zeaxanthin–bismelatonin conjugate (**14**) (**a**) in THF and in THF-PBS mixtures (1:1, 1:3); (**b**) in DMSO and in DMSO-PBS mixtures (5 µM, 3.75 µM, 2.5 µM, 1.25 µM, 0.625 µM and 0.3125 µM).

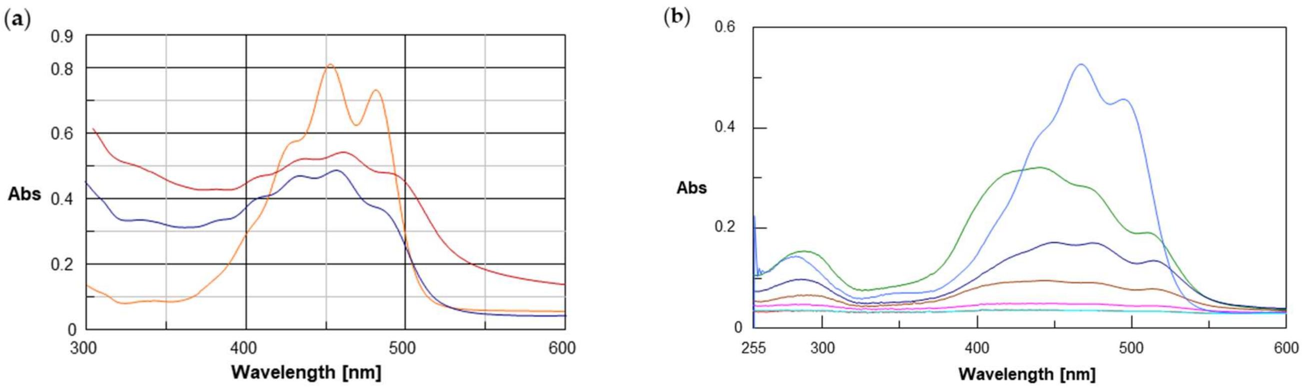

Figure S9. UV-spectra of β-cryptoxanthin–melatonin conjugate (**15**) (**a**) in THF and in THF-PBS mixtures (1:1, 1:3); (**b**) in DMSO and in DMSO-PBS mixtures (5 µM, 3.75 µM, 2.5 µM, 1.25 µM, 0.625 µM and 0.3125 µM).

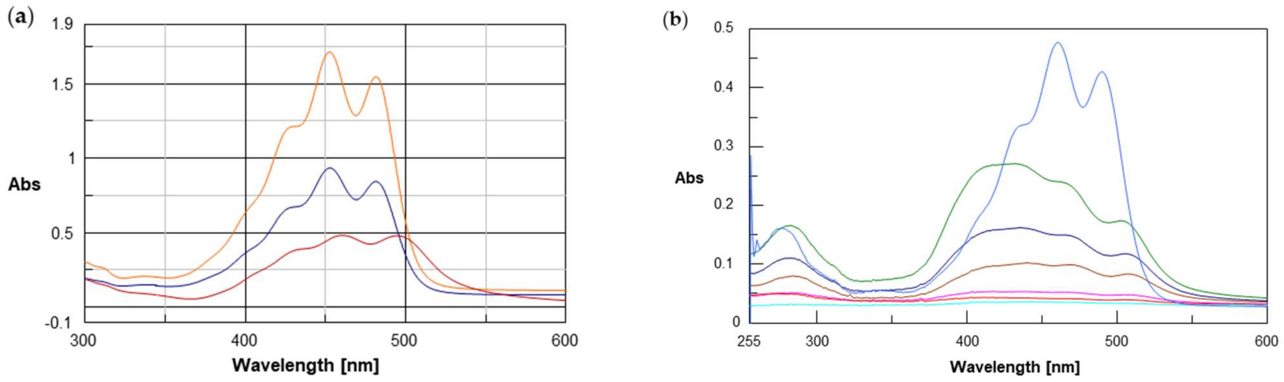

Figure S10. UV-spectra of lutein–bismelatonin conjugate (**16**) (**a**) in THF and in THF-PBS mixtures (1:1, 1:3); (**b**) in DMSO and in DMSO-PBS mixtures (5 μM, 3.75 μM, 2.5 μM, 1.25 μM, 0.625 μM and 0.3125 μM).

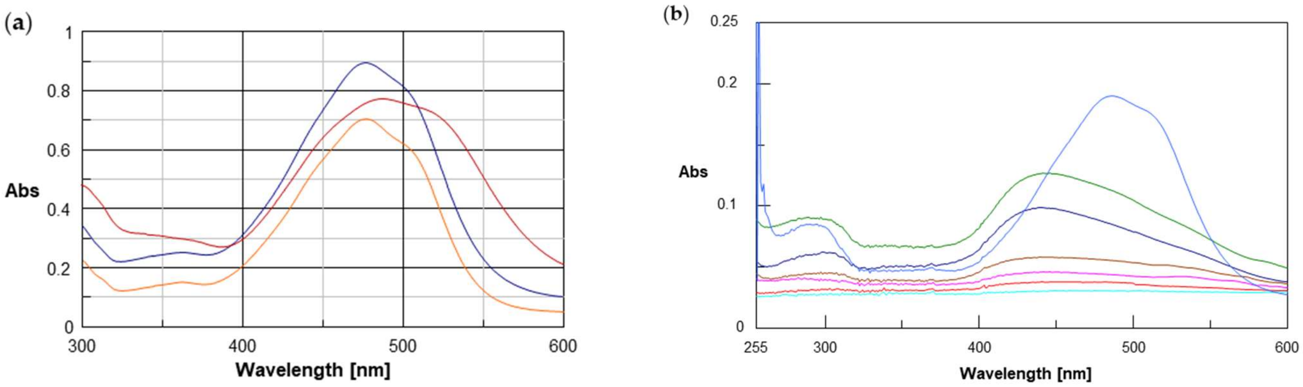

Figure S11. UV-spectra of capsanthin–bismelatonin conjugate (**17**) (**a**) in THF and in THF-PBS mixtures (1:1, 1:3); (**b**) in DMSO and in DMSO-PBS mixtures (5 μM, 3.75 μM, 2.5 μM, 1.25 μM, 0.625 μM and 0.3125 μM).

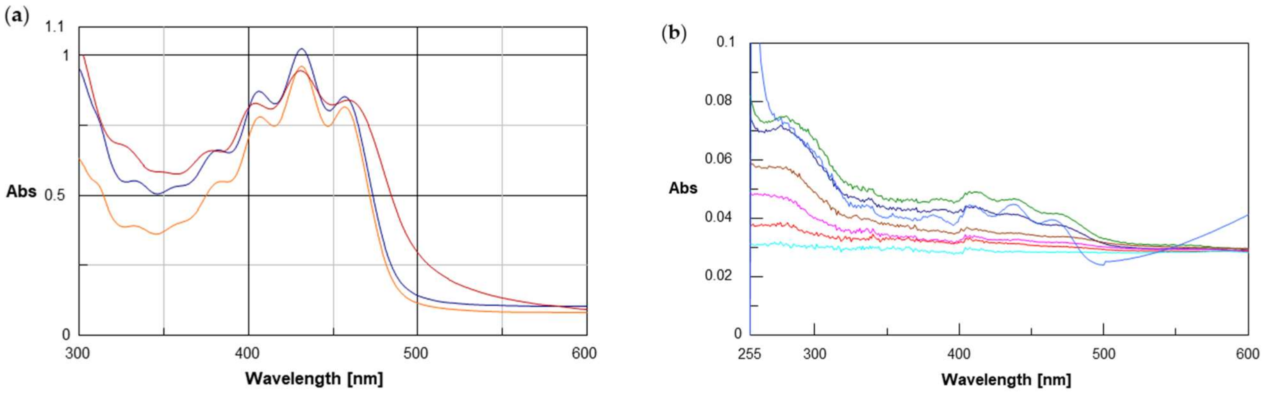

Figure S12. UV-spectra of 8′-apo-β-carotenol–melatonin conjugate (**18**) (**a**) in THF and in THF-PBS mixtures (1:1, 1:3); (**b**) in DMSO and in DMSO-PBS mixtures (5 μM, 3.75 μM, 2.5 μM, 1.25 μM, 0.625 μM and 0.3125 μM).

2. Dinamic Light Scattering

The number-weighted size distribution was used to determine the hydrodynamic diameter (mean size) of the aggregates.

6 (8'-apo-β-carotenol succinate)

conc. (μM)	mean size (nm)	st dev.	PDI	zeta potential (mV)
0.3125	303.50	35.51	0.0137	−8.2
0.625	395.10	84.32	0.0455	−10.2
1.25	221.50	32.73	0.0218	−17.7
2.5	26.74	3.841	0.0206	−30.5
3.75	152.60	24.96	0.0268	−12.3
5	144.20	20.59	0.0204	−27.5

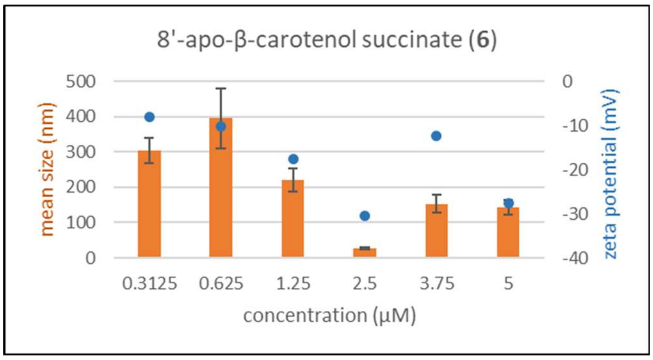

7 (zeaxanthin bissuccinate)

conc (μM)	mean size (nm)	st dev	PDI	zeta potential (mV)
0.3125	1050.00	211.70	0.0407	27.5
0.625	429.30	54.63	0.0162	23.4
1.25	544.50	93.34	0.0294	21.1
2.5	195.60	27.81	0.0202	17.9
3.75	44.78	7.32	0.0267	20.0
5	238.20	39.12	0.0270	20.1

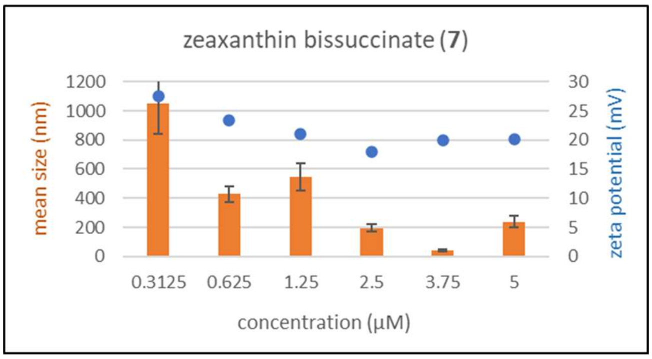

8 (cryptoxantin succinate)

conc (μM)	mean size (nm)	st dev	PDI	zeta potential (mV)
0.3125	11.04	1.43	0.0168	34.1
0.625	25.68	4.50	0.0307	27.5
1.25	31.94	8.59	0.0723	21
2.5	34.03	6.46	0.0360	17.6
3.75	468.90	53.65	0.0131	2.55
5	14.19	2.61	0.0339	−0.01

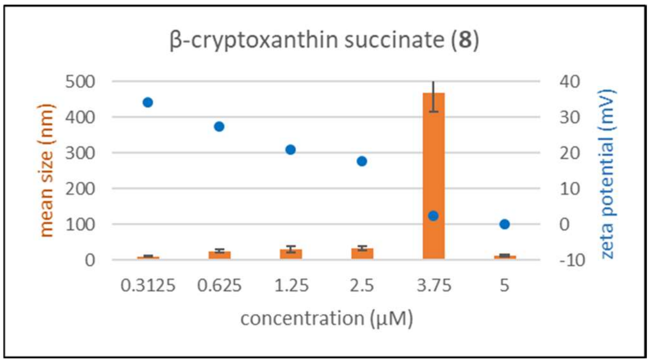

9 (capsanthin bissuccinate)

conc (μM)	mean size (nm)	st dev	PDI	zeta potential (mV)
0.3125	241.5	32.96	0.0186	22.6
0.625	10.71	1.35	0.0159	20.1
1.25	28.78	5.42	0.0354	31.0
2.5	256.4	26.59	0.0108	51.3
3.75	256.4	26.59	0.0108	2.7
5	19.31	3.34	0.0298	0.0

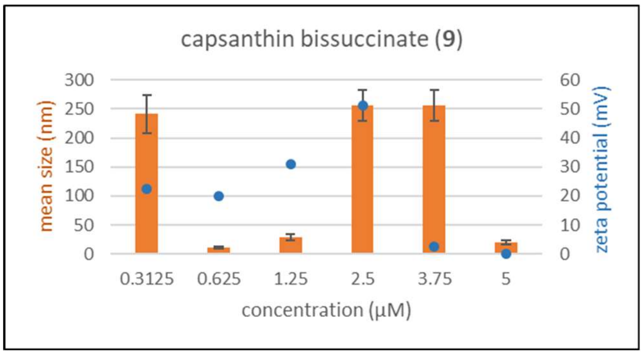

10 (lutein bissuccinate)

conc (μM)	mean size (nm)	st dev	PDI	zeta potential (mV)
0.3125	453.5	69.55	0.0235	103.0
0.625	215.4	38.51	0.0320	44.4
1.25	263.3	64.95	0.0608	89.6
2.5	123.1	12.77	0.0108	0.0
3.75	143.8	15.79	0.0121	0.0
5	29.2	4.61	0.0249	20.3

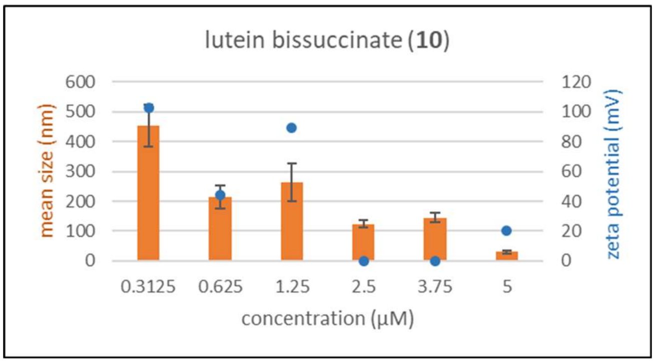

11 (zeaxanthin monosuccinate)

conc (μM)	mean size (nm)	st dev	PDI	zeta potential (mV)
0.3125	30.98	4.96	0.0256	51.7
0.625	277.80	45.12	0.0264	123.0
1.25	25.09	4.42	0.0310	80.5
2.5	32.93	6.75	0.0420	80.6
3.75	17.71	6.52	0.1357	77.3
5	150.00	25.87	0.0297	−23.5

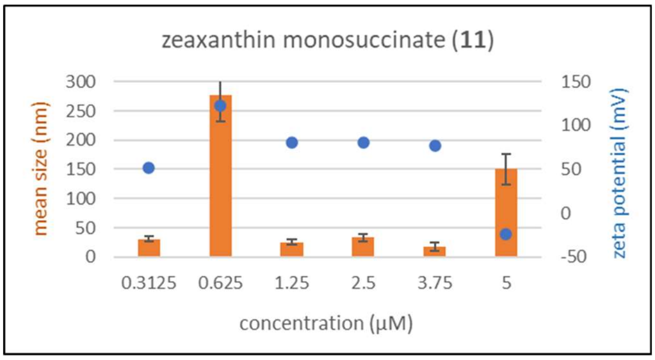

13 (zeaxanthin monomelatonin)

conc (μM)	mean size (nm)	st dev	PDI	zeta potential (mV)
0.3125	388.20	71.14	0.0336	16.0
0.625	551.20	111.60	0.0410	15.4
1.25	364.50	59.90	0.0270	2.2
2.5	638.90	94.06	0.0217	2.2
3.75	79.01	8.35	0.0112	0.7
5	565.30	99.67	0.0311	1.1

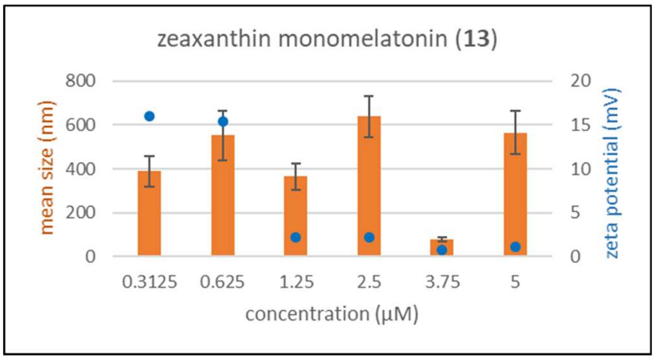

14 (zeaxanthin bismelatonin)

conc (μM)	mean size (nm)	st dev	PDI	zeta potential (mV)
0.3125	- *	-	-	−0.7
0.625	- *	-	-	−0.7
1.25	- *	-	-	−1.0
2.5	137.50	16.65	0.0147	−0.4
3.75	91.77	9.52	0.0108	−0.2
5	705.10	181.00	0.0659	0.0

* The size of the particles were too large for DLS

15 (β-cryptoxantin melatonin)

conc (μM)	mean size (nm)	st dev	PDI	zeta potential (mV)
0.3125	29.79	5.62	0.0356	−13.4
0.625	31.22	10.95	0.1230	−23.5
1.25	74.91	24.29	0.1051	−20.8
2.5	305.10	123.90	0.1649	−20.4
3.75	826.80	239.60	0.0840	−11.8
5	615.10	193.70	0.0992	−15.7

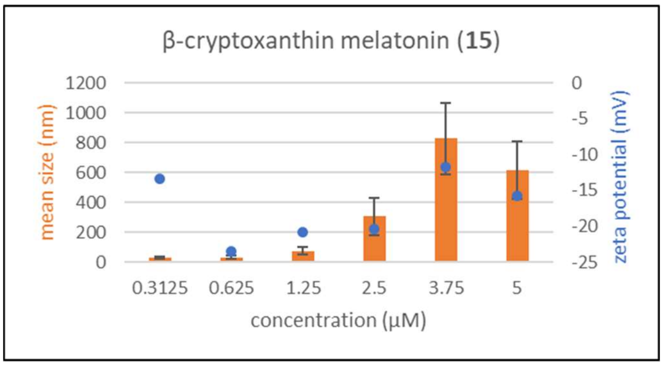

16 (lutein bismelatonin)

conc (μM)	mean size (nm)	st dev	PDI	zeta potential (mV)
0.3125	131.20	58.99	0.2022	−12.2
0.625	51.10	13.26	0.0673	−11.7
1.25	142.50	14.79	0.0108	−12.5
2.5	91.19	36.31	0.1585	−27.5
3.75	39.28	13.77	0.1229	−1.4
5	43.14	7.01	0.0264	−0.5

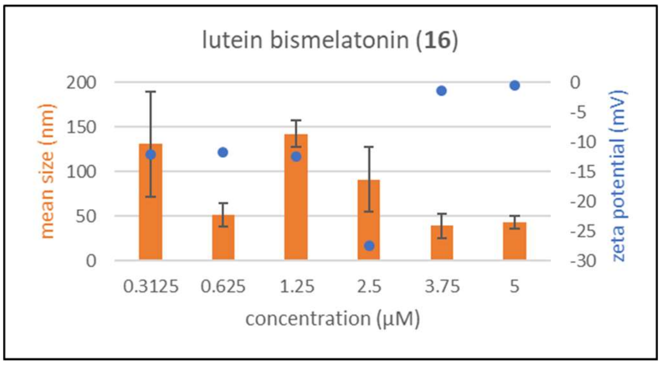

17 (capsanthin bismelatonin)

conc (μM)	mean size (nm)	st dev	PDI	zeta potential (mV)
0.3125	24.42	4.94	0.0408	−31.0
0.625	43.47	15.30	0.1239	−22.9
1.25	101.60	37.50	0.1362	−21.1
2.5	226.30	91.92	0.1650	−22.2
3.75	58.55	24.09	0.1693	−27.5
5	236.60	84.43	0.1273	−27.0

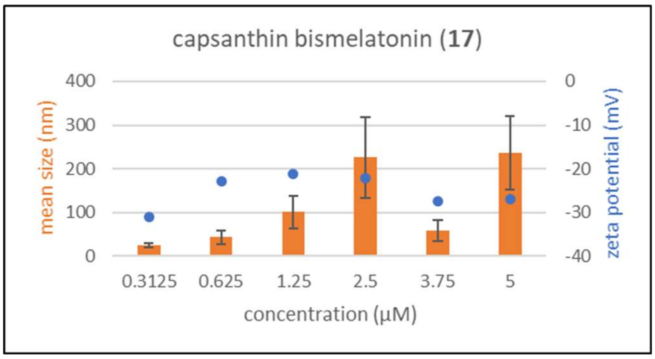

18 (8'-apo-β-carotenol melatonin)

conc (μM)	mean size (nm)	st dev	PDI	zeta potential (mV)
0.3125	148.60	20.94	0.0199	−22.0
0.625	212.00	42.26	0.0397	−22.8
1.25	127.50	34.35	0.0726	−24.5
2.5	93.29	32.70	0.1229	−29.5
3.75	182.80	74.53	0.1662	−31.4
5	201.10	85.03	0.1788	−37.5

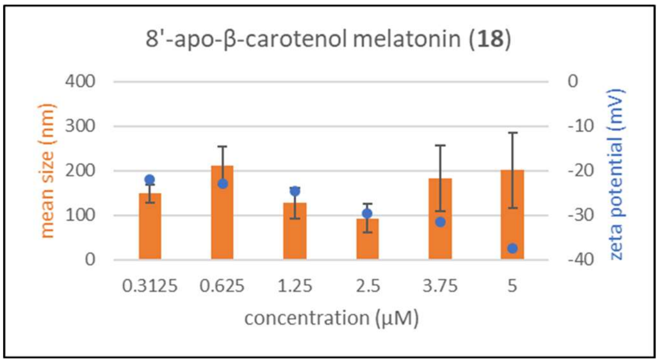

3. Statistical Analysis of the Antioxidant Measurements by ABTS Method

All experiments were carried out in triplicate. For the comparison of the means, one-way ANOVA with Student-Newman–Keuls post-hoc test was calculated, and to analyze the homogeneity of variance, Levene statistics were implemented by using SPSS 26.0 (SPSS, Chicago, IL, USA). A difference was considered statistically significant at $p < 0.05$.

As the carotenoids and the conjugates are poorly soluble in water, they were dissolved first either in small amounts of freshly distilled tetrahydrofuran (THF) or in dimethylsulfoxide (DMSO), and these stock solutions were diluted with phosphate-buffered saline (PBS) to the final nominal concentration.

Table S1. TEAC values and their standard deviations determined by ABTS method

Su#bstance	TEAC from THF	SD from THF	TEAC from DMSO	SD from DMSO
Zeaxanthin (**2**)	2.016	0.021	2.70	0.04
β-cryptoxanthin (**3**)	2.062	0.027	1.61	0.03
Capsanthin (**4**)	1.036	0.008	0.62	0.01
Lutein (**5**)	1.571	0.018	1.02	0.00
8'-Apo-β-carotenal	0.953	0.016	1.19	0.01
8'-Apo-β-carotenol succinate (**6**)	1.913	0.022	1.19	0.01
Zeaxanthin bissuccinate (**7**)	2.090	0.037	0.86	0.00
β-Cryptoxanthin succinate (**8**)	1.827	0.041	1.13	0.02
Capsanthin bissuccinate (**9**)	2.297	0.065	1.66	0.02
Lutein bissuccinate (**10**)	2.356	0.041	1.49	0.01
Zeaxanthin monosuccinate (**11**)	1.056	0.030	0.81	0.03
Zeaxanthin monomelatonin (**13**)	1.434	0.026	1.34	0.07
Zeaxanthin bismelatonin (**14**)	2.080	0.023	2.79	0.05
β-Cryptoxanthin melatonin (**15**)	1.777	0.014	1.13	0.02
Lutein bismelatonin (**16**)	2.933	0.003	2.40	0.04
Capsanthin bismelatonin (**17**)	1.519	0.043	2.53	0.01
8'-Apo-β-carotenol melatonin (**18**)	2.891	0.009	3.00	0.05
Zeaxanthin-melatonin 1:2 mixture	12.039	0.044	13.72	0.02
Melatonin in EtOH	5.462	0.040	5.81	0.07

Effect of the modification of the carotenoids:

Derivatives of zeaxanthin (**2, 7, 11, 13, 14**)*:*

From THF: There was a significant difference among the TEAC values, F (6, 14) = 4936.591, $p < 0.001$ (1.890E-22). Post-hoc testing revealed significant differences among the derivatives of zeaxanthin in phosphate buffer but significant a difference cannot be identified in case of zeaxanthin (**2**), zeaxanthin bissuccinate (**7**) and zeaxanthin bismelatonin derivative (**14**).

From DMSO: There was a significant difference among the TEAC values, F (6, 14) = 33202.050, $p < 0.001$ (3.046E-28). Post-hoc testing revealed significant differences among the derivatives of zeaxanthin in phosphate buffer but a significant difference cannot be identified in case of zeaxanthin monosuccinate (**11**) and zeaxanthin disuccinate (**7**).

Derivatives of β-cryptoxanthin (**3, 8, 15**)

From THF: There was a significant difference among the TEAC values, F (3, 8) = 5902.737, $p < 0.001$ (1.023E-13). Post-hoc testing showed a significant differences among the derivatives of β-cryptoxanthin.

From DMSO: There was a significant difference among the TEAC values, F (3, 8) = 5913.638, $p < 0.001$ (1.016E-13). Post-hoc testing showed significant differences among the derivatives of β-cryptoxanthin.

Derivatives of capsanthin (**4, 9, 17**)

From THF: There was a significant difference among the TEAC values, F (3, 8) = 6221.102, $p < 0.001$ (8.293E-14). Post-hoc testing showed significant differences among the derivatives of capsanthin.

From DMSO: There was a significant difference among the TEAC values, F (3, 8) = 11858.312, $p < 0.001$ (6.287E-15). Post-hoc testing showed significant differences among the derivatives of capsanthin.

Derivatives of lutein (**5, 10, 16**)

From THF: There was a significant difference among the TEAC values, F (3, 8) = 9432.513, $p < 0.001$ (1.570E-14). Post-hoc testing showed significant differences among the derivatives of lutein.

From DMSO: There was a significant difference among the TEAC values, F (3, 8) = 8570.642, $p < 0.001$ (2.303E-14). Post-hoc testing showed significant differences among the derivatives of lutein.

Derivatives of 8′-apo-β-carotenol (**6, 18**)

From THF: There was a significant difference among the TEAC values, F (3, 8) = 18869.485, $p < 0.001$ (9.810E-16). Post-hoc testing showed significant differences among the derivatives of 8'-apo-β-carotenal.

From DMSO: There was a significant difference among the TEAC values, F (3, 8) = 7285.815, $p < 0.001$ (4.409E-14). Post-hoc testing showed significant differences among the derivatives of 8'-apo-β-carotenal in phosphate buffer, but there was no statistical difference between 8'-apo-β-carotenal and 8'-Apo-β-carotenol succinate (**6**).

4. Statistical Analysis of the Antioxidant Measurements by FRAP Method

All experiments were carried out in triplicate. A difference was considered statistically significant at $p < 0.05$. The carotenoids were dissolved first either in small amounts of freshly distilled tetrahydrofuran (THF), and these stock solutions were diluted with acetate buffer to the final nominal concentration.

Table S2. FRAP values and their standard deviations.

**Substance**	**FRAP**	**SD**
Zeaxanthin (**2**)	0.351	0.009
β-cryptoxanthin (**3**)	0.441	0.012
Capsanthin (**4**)	0.197	0.001
Lutein (**5**)	0.161	0.006
8'-apo-β-carotenal	0.443	0.005
8'-Apo-β-carotenol succinate (**6**)	0.276	0.003
Zeaxanthin bissuccinate (**7**)	0.155	0.005
β-Cryptoxanthin succinate (**8**)	0.181	0.003
Capsanthin bissuccinate (**9**)	0.189	0.008
Lutein bissuccinate (**10**)	0.184	0.005
Zeaxanthin monosuccinate (**11**)	0.145	0.007
Zeaxanthin monomelatonin (**13**)	0.152	0.003
Zeaxanthin bismelatonin (**14**)	0.206	0.005
β-Cryptoxanthin melatonin (**15**)	0.154	0.005
Lutein bismelatonin (**16**)	0.460	0.009
Capsanthin bismelatonin (**17**)	0.629	0.015
8'-Apo-β-carotenol melatonin (**18**)	0.123	0.001
Zeaxanthin–melatonin 1:2 mixture	0.302	0.002
Melatonin in EtOH	0.038	0.002

Effect of the modification of the carotenoids:

Derivatives of zeaxanthin (**2, 7, 11, 13, 14**)

There was a significant difference among the FRAP, $F (6, 14) = 1181.110$, $p < 0.001$ (4.155E-18). Post-hoc testing revealed significant differences among the derivatives of *zeaxanthin, but* there is no statistically significantly difference among zeaxanthin monosuccinate (**11**), zeaxanthin disuccinate (**7**) and zeaxanthin dimelatonin (**14**).

Derivatives of β-cryptoxanthin (**3, 8, 15**)

There was a significant difference among the FRAP, $F (3, 8) = 1916.360$, $p < 0.001$ (9.171E-12). Post-hoc testing revealed significant differences among the derivatives of β-cryptoxanthin.

Derivatives of capsanthin (**4, 9, 17**)

There was a significant difference among the FRAP, $F (3, 8) = 2673.149$, $p < 0.001$ (2.427E-12). Post-hoc testing revealed significant differences among the derivatives of capsanthin, but there is no statistically significantly difference between capsanthin (**4**) and capsanthin disuccinate (**9**).

Derivatives of lutein (**5, 10, 16**)

There was a significant difference among the FRAP, $F (3, 8) = 2470.847$, $p < 0.001$ (3.323E-12). Post-hoc testing revealed significant differences among the derivatives of lutein.

Derivatives of 8'-apo-β-carotenol (**6, 18**)

There was a significant difference among the FRAP, $F (3, 8) = 9353.907$, $p < 0.001$ (1.624E-14). Post-hoc testing revealed significant differences among the derivatives of 8'-apo-β-carotenol.

5. ^{1}H and ^{13}C-dept NMR Spectra of the Bismelatonin Conjugate of Zeaxanthin (14)

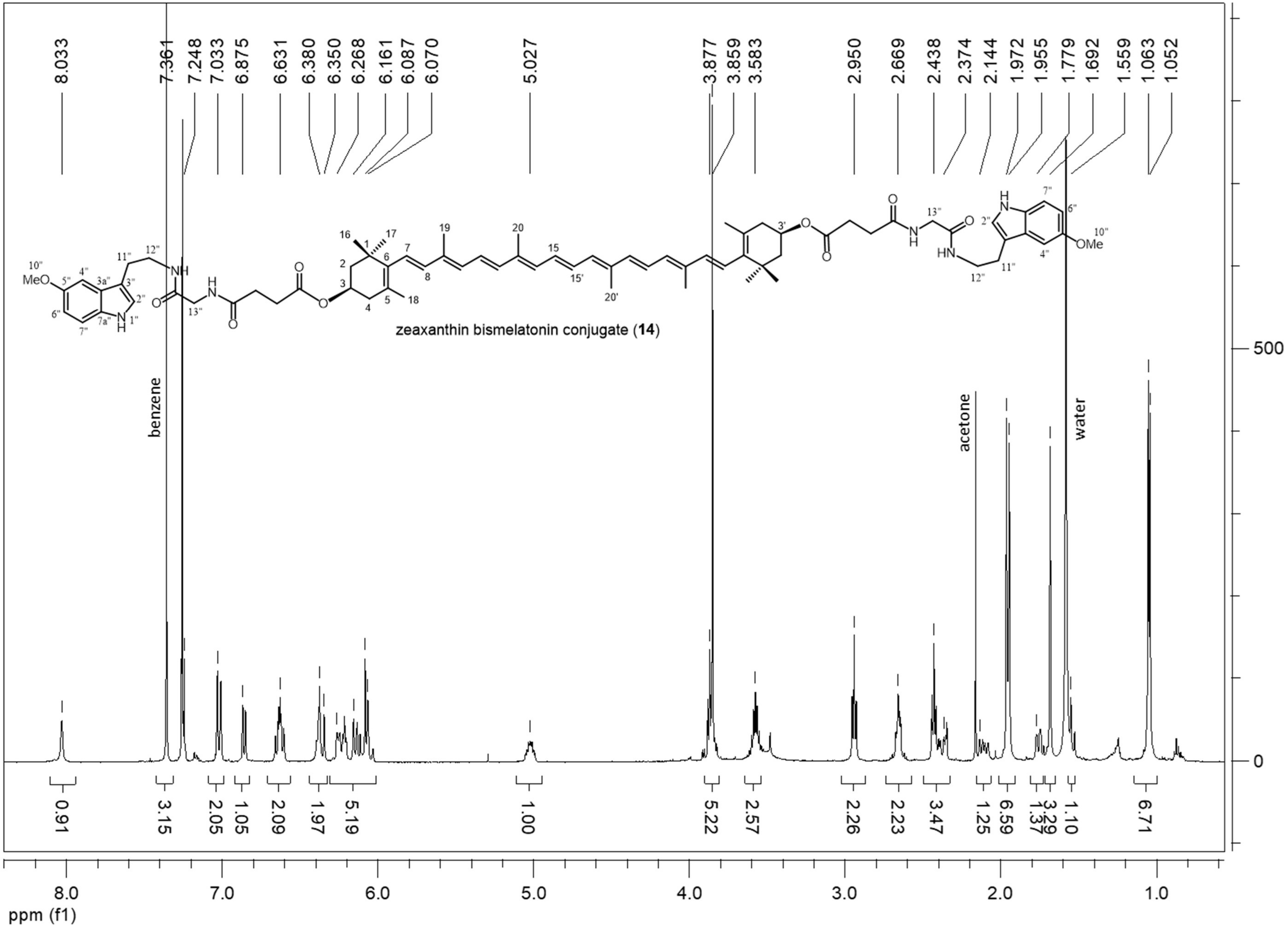

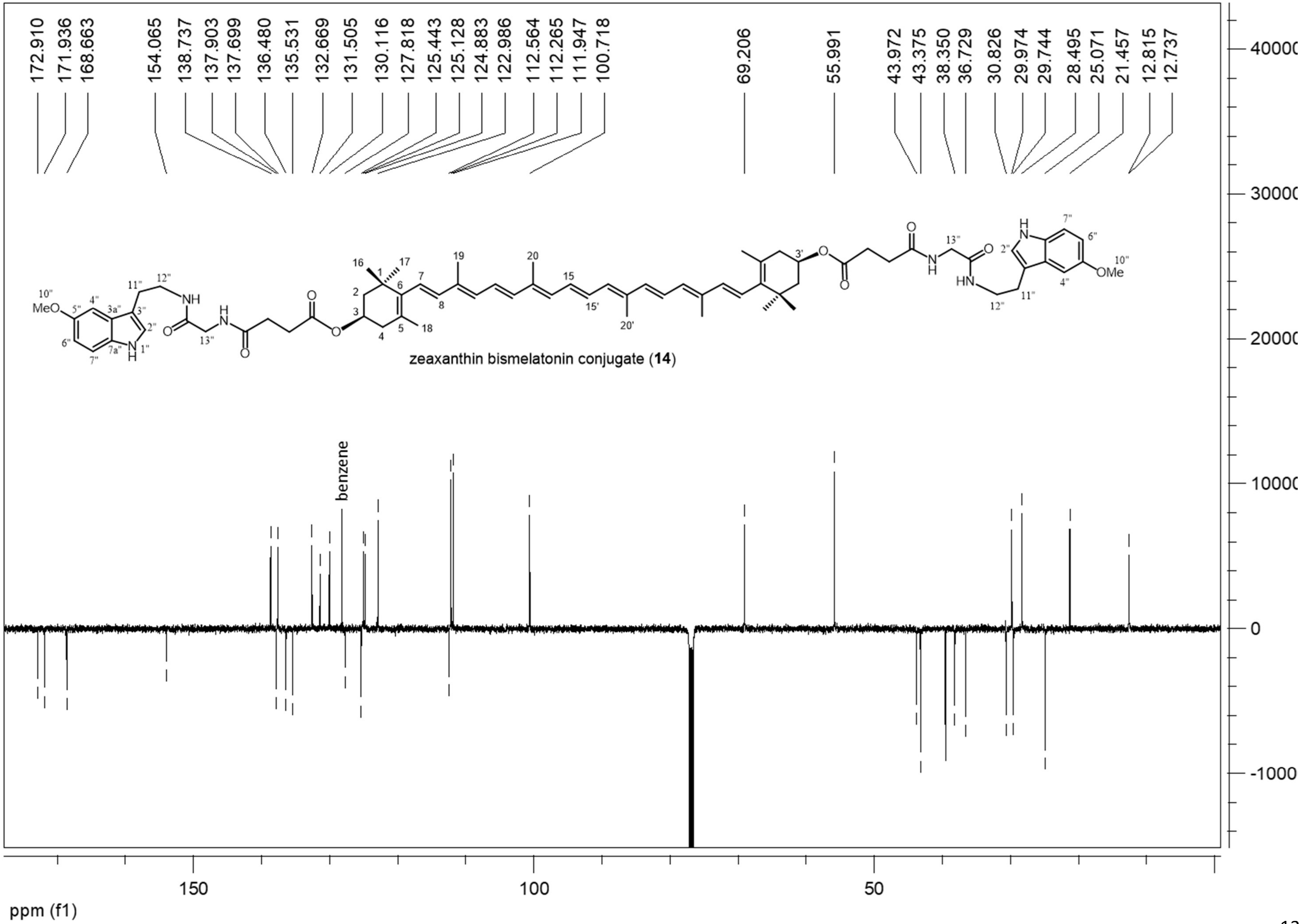
172.910
171.936
168.663
154.065
138.737
137.903
137.699
136.480
135.531
132.669
131.505
130.116
127.818
125.443
125.128
124.883
122.986
112.564
112.265
111.947
100.718
69.206
55.991
43.972
43.375
38.350
36.729
30.826
29.974
29.744
28.495
25.071
21.457
12.815
12.737
zeaxanthin bismelatonin conjugate (14)
benzene
40000
30000
20000
10000
0
-1000
150
100
50
ppm (f1)

6. HPLC Chromatograms of the Synthesized Compounds

The purity of the synthesized carotenoid derivatives was determined by the following HPLC methods: *Method I* is for the carotenoid succinates (compounds **6–10**); *Method II* is for the melatonin conjugates (compounds **13–18**).

Instrumentation:
The HPLC analysis was performed with a Dionex P680 quaternary analytical pump, a Dionex PDA 100 UV/VIS detector (Thermo Fisher Scientific, Inc., Waltham, MA) with Chromeleon 6.8 software and a column temperature control module. The separations were performed on a LiChrospher® 100 RP-18e (particle size: 5 μm, pore size: 100 Å) (Merck KGaA, Darmstadt, Germany) column (250 mm × 4.00 mm) with gradient elution.

Method I: (for the carotenoid succinates **6–10**)
Eluent A: 1% acetic acid in distilled water, Eluent B: Methanol
A linear gradient was implemented: from 93% B to 98% B in 35 minutes, returns to 93% B in 2 min.
Flow rate: 1.5 mL/min, temperature: 22 °C, detection wavelength: λ = 450 nm

Method II: (for the melatonin conjugates **13–18**)
Eluents: A: 12% H_2O / 88% MeOH
B: 100% MeOH
C: 50% Acetone / 50% MeOH
Gradient program:
0–8 min: from 100% A to 50% A/50% B,
8–15 min: to 100% B
15–17 min: 100% B,
17–24 min: to 100% C,
24–31 min: 100% C,
31–33 min: to 100% B, 0% C
33–34 min: 100% B,
34–35 min 80% A, 20% B, 0% C
Flow rate: 1.25 mL/min, temperature: 22 °C, detection wavelength: λ = 450 nm

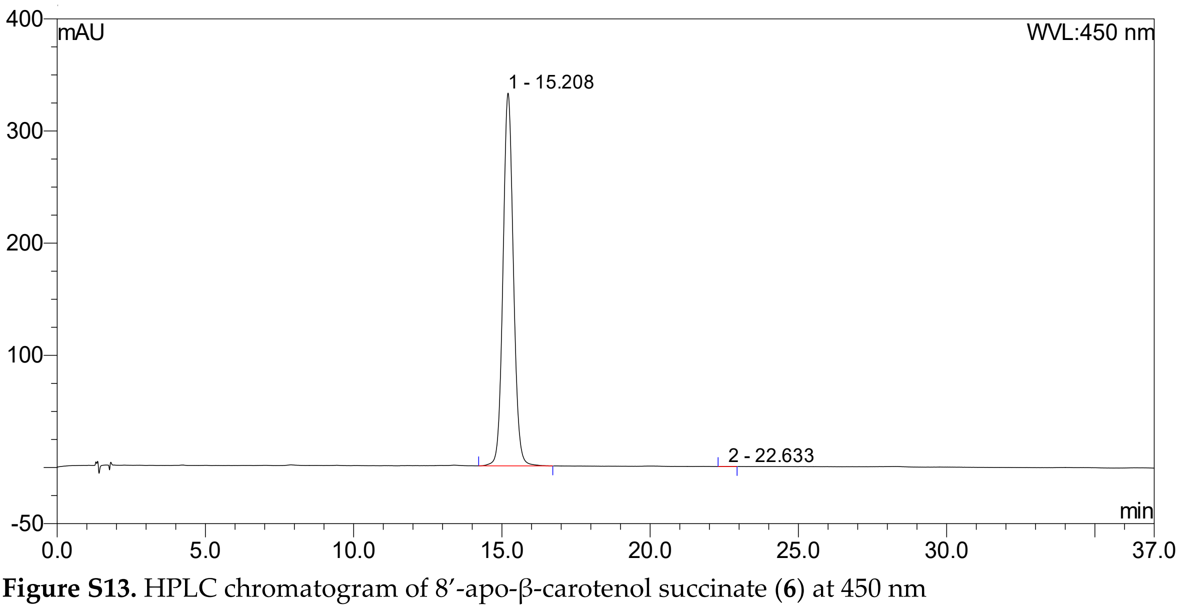

Figure S13. HPLC chromatogram of 8′-apo-β-carotenol succinate (**6**) at 450 nm

No.	Ret.Time min	Peak Name	Height mAU	Area mAU*min	Rel.Area %	Amount	Type
1	15,21	n.a.	332,369	134,838	99,99	n.a.	BMB
2	22,63	n.a.	0,092	0,020	0,01	n.a.	BMB*
**Total:**			332,460	134,858	100,00	0,000	

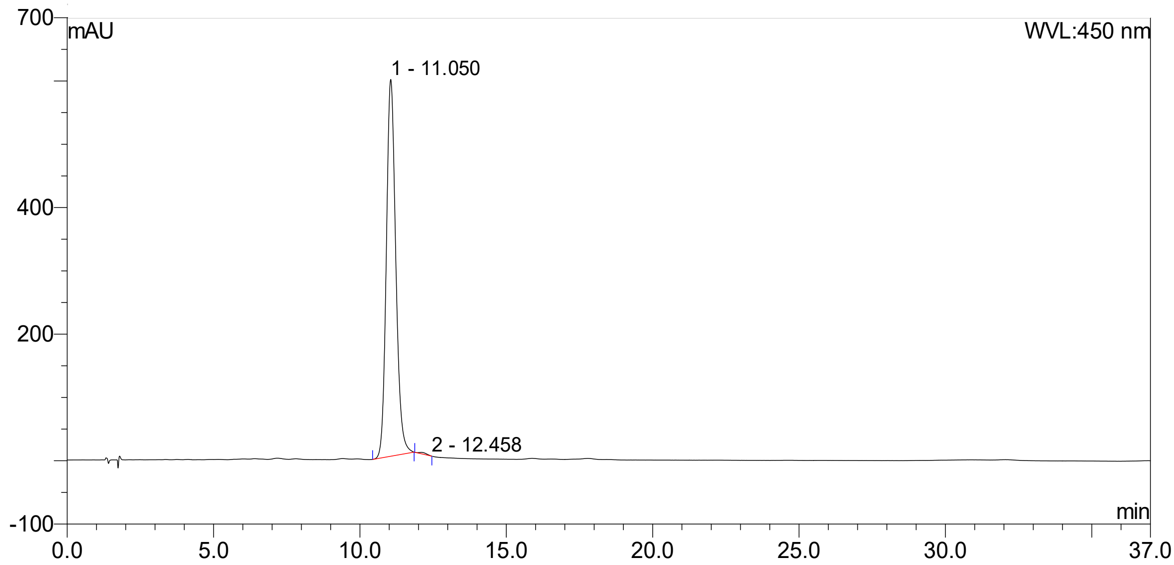

Figure S14. HPLC chromatogram of zeaxantin bissuccinate (**7**) at 450 nm

No.	Ret.Time min	Peak Name	Height mAU	Area mAU*min	Rel.Area %	Amount	Type
1	11,05	n.a.	595,445	222,336	99,66	n.a.	BMB*
2	12,46	n.a.	0,000	0,753	0,34	n.a.	BMB*
**Total:**			595,445	223,089	100,00	0,000	

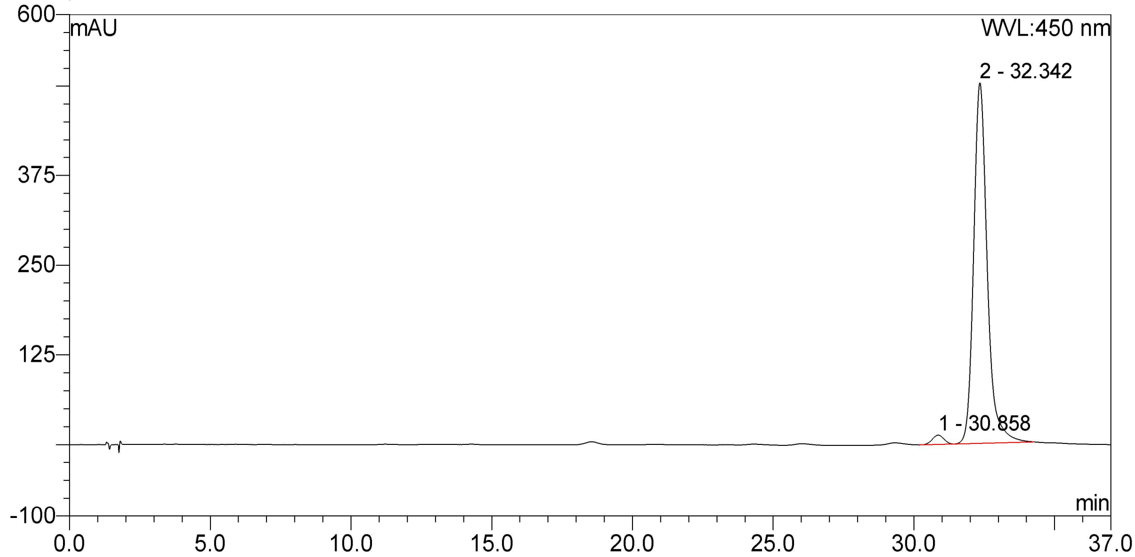

Figure S15. HPLC chromatogram of β-cryptoxanthin succinate (**8**) at 450 nm

No.	Ret.Time min	Peak Name	Height mAU	Area mAU*min	Rel.Area %	Amount	Type
1	30,86	n.a.	12,875	6,155	2,15	n.a.	BM *
2	32,34	n.a.	502,810	280,640	97,85	n.a.	MB*
**Total:**			515,684	286,795	100,00	0,000	

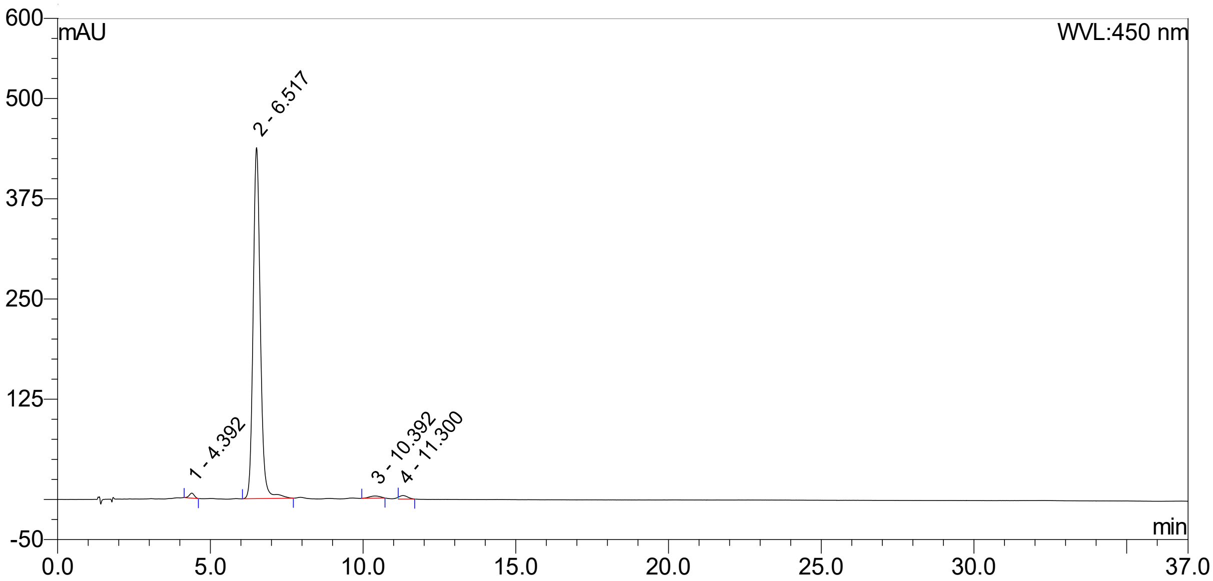

Figure S16. HPLC chromatogram of capsanthin bissuccinate (**9**) at 450 nm

No.	Ret.Time min	Peak Name	Height mAU	Area mAU*min	Rel.Area %	Amount	Type
1	4,39	n.a.	6,164	1,190	0,97	n.a.	BMB*
2	6,52	n.a.	437,734	119,498	97,08	n.a.	BMB
3	10,39	n.a.	2,653	1,065	0,87	n.a.	BMB*
4	11,30	n.a.	4,355	1,340	1,09	n.a.	MB*
**Total:**			**450,906**	**123,093**	**100,00**	**0,000**	

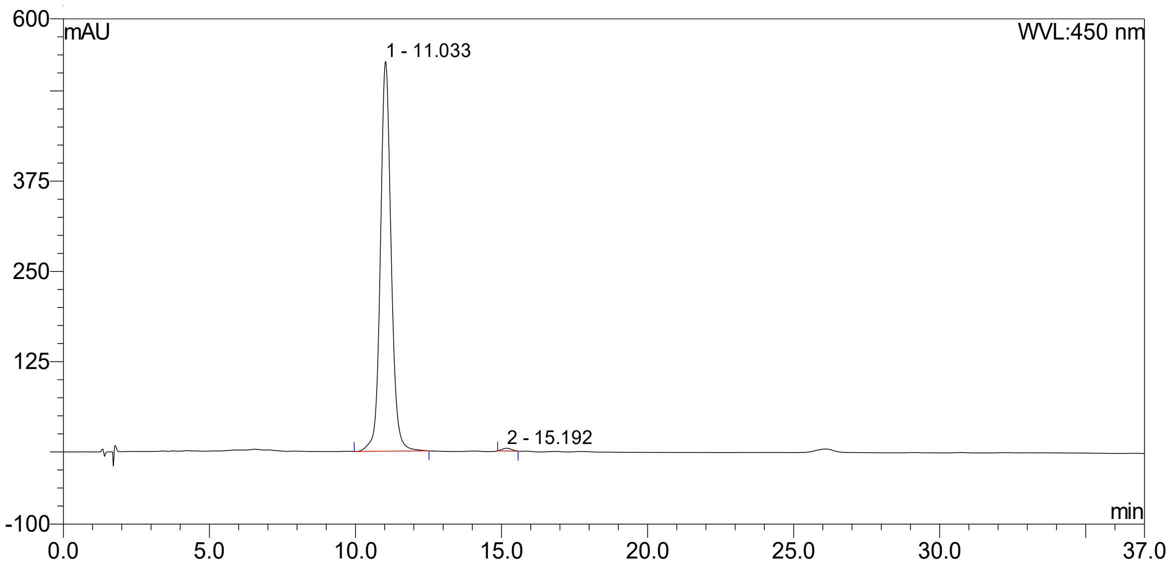

Figure S17. HPLC chromatogram of lutein bissuccinate (**10**) at 450 nm

No.	Ret.Time min	Peak Name	Height mAU	Area mAU*min	Rel.Area %	Amount	Type
1	11,03	n.a.	539,837	229,963	99,39	n.a.	BMB*
2	15,19	n.a.	3,817	1,413	0,61	n.a.	BMB*
**Total:**			543,654	231,375	100,00	0,000	

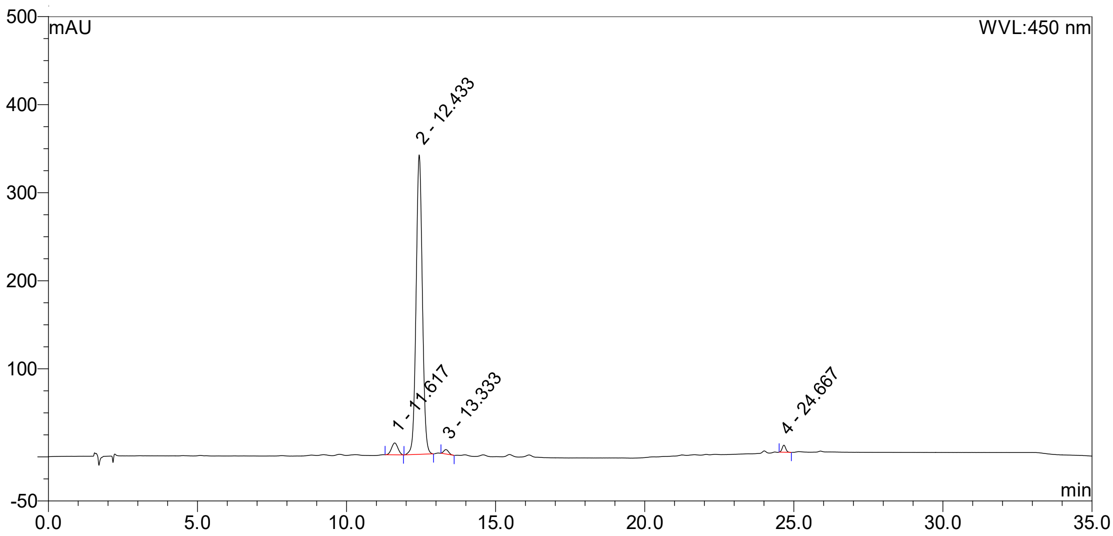

Figure S18. HPLC chromatogram of zeaxanthin–monomelatonin conjugate (**13**) at 450 nm

No.	Ret.Time min	Peak Name	Height mAU	Area mAU*min	Rel.Area %	Amount	Type
1	11,62	n.a.	13,469	3,334	3,79	n.a.	BMB
2	12,43	n.a.	340,117	82,648	93,90	n.a.	BMB
3	13,33	n.a.	5,052	0,925	1,05	n.a.	BMB*
4	24,67	n.a.	8,050	1,111	1,26	n.a.	BMB*
**Total:**			366,687	88,018	100,00	0,000	

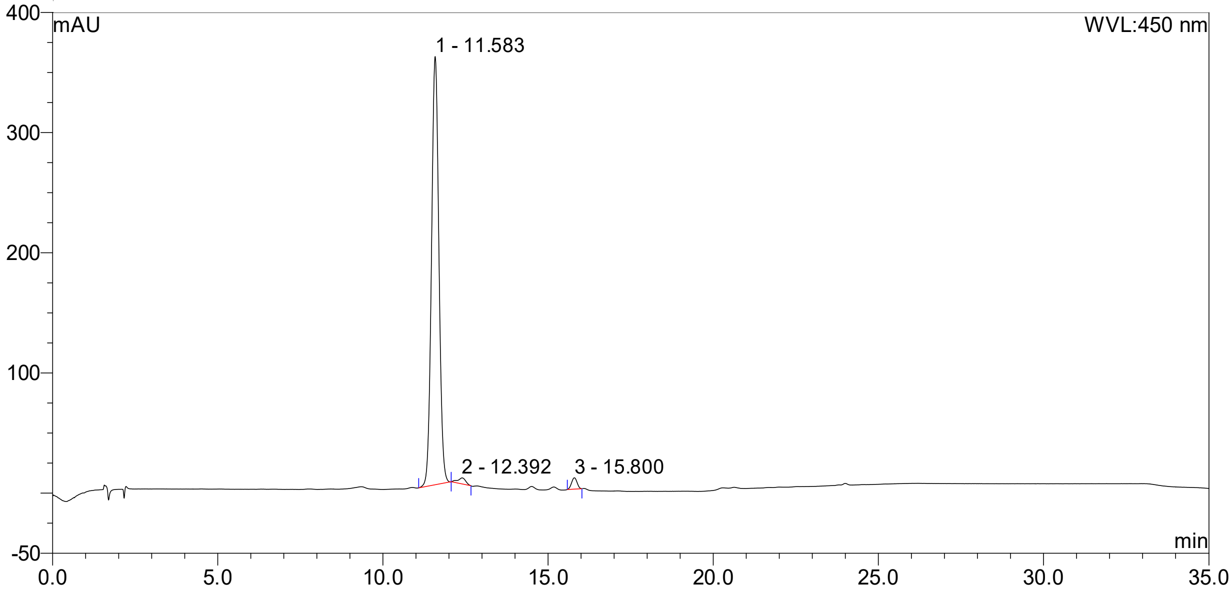

Figure S19. HPLC chromatogram of zeaxanthin–bismelatonin conjugate (**14**) at 450 nm

No.	Ret.Time min	Peak Name	Height mAU	Area mAU*min	Rel.Area %	Amount	Type
1	11,58	n.a.	356,361	92,281	96,65	n.a.	BMB*
2	12,39	n.a.	4,994	1,386	1,45	n.a.	bMB*
3	15,80	n.a.	9,421	1,810	1,90	n.a.	BMB*
**Total:**			370,775	95,477	100,00	0,000	

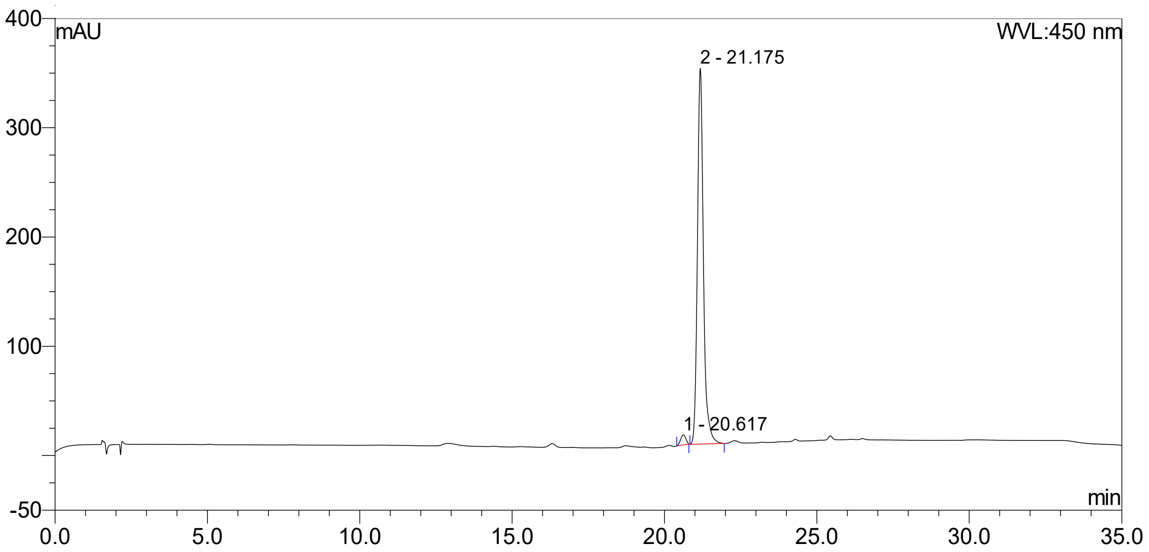

Figure S20. HPLC chromatogram of β-cryptoxanthin–melatonin conjugate (**15**) at 450 nm

**No.**	**Ret.Time min**	**Peak Name**	**Height mAU**	**Area mAU*min**	**Rel.Area %**	**Amount**	**Type**
1	20,62	n.a.	9,167	1,824	2,28	n.a.	BMB*
2	21,18	n.a.	343,895	78,303	97,72	n.a.	BMB
**Total:**			353,062	80,127	100,00	0,000	

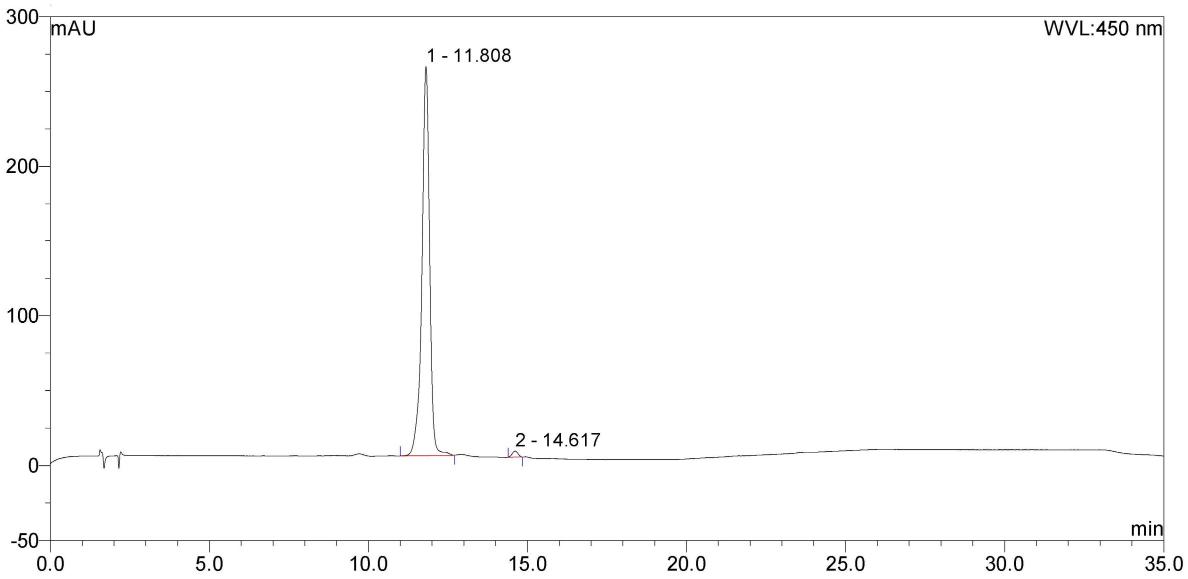

Figure S21. HPLC chromatogram of lutein–bismelatonin conjugate (**16**) at 450 nm

**No.**	**Ret.Time min**	**Peak Name**	**Height mAU**	**Area mAU*min**	**Rel.Area %**	**Amount**	**Type**
1	11,81	n.a.	260,073	73,076	98,86	n.a.	BMB
2	14,62	n.a.	4,061	0,844	1,14	n.a.	BMB*
**Total:**			264,134	73,920	100,00	0,000	

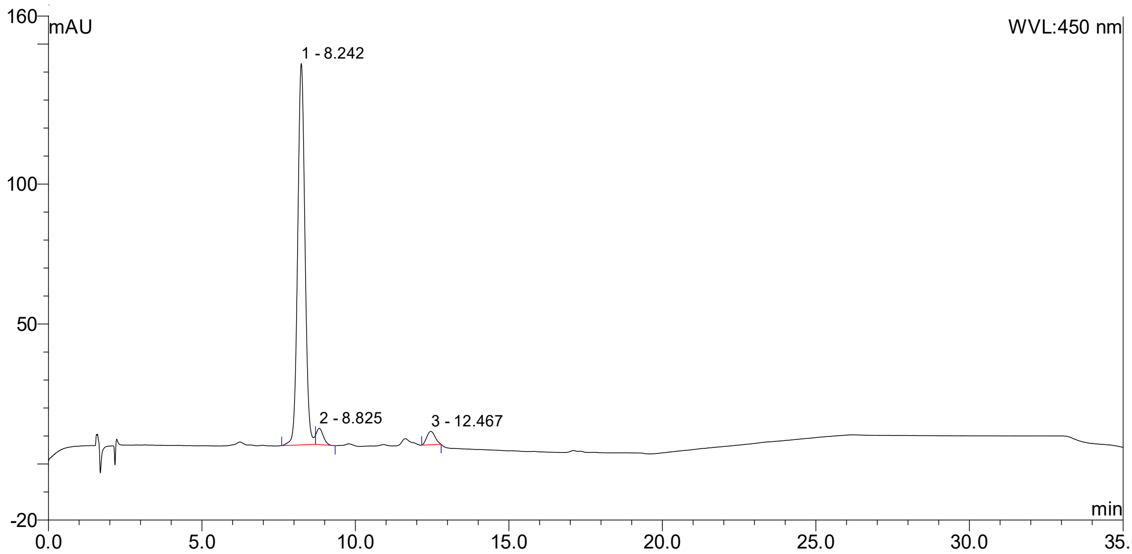

Figure S22. HPLC chromatogram of capsanthin bismelatonin–conjugate (**17**) at 450 nm

**No.**	**Ret.Time min**	**Peak Name**	**Height mAU**	**Area mAU*min**	**Rel.Area %**	**Amount**	**Type**
1	8,24	n.a.	136,202	37,437	92,63	n.a.	BM *
2	8,83	n.a.	5,814	1,481	3,67	n.a.	MB*
3	12,47	n.a.	4,744	1,496	3,70	n.a.	BMB*
**Total:**			146,760	40,414	100,00	0,000	

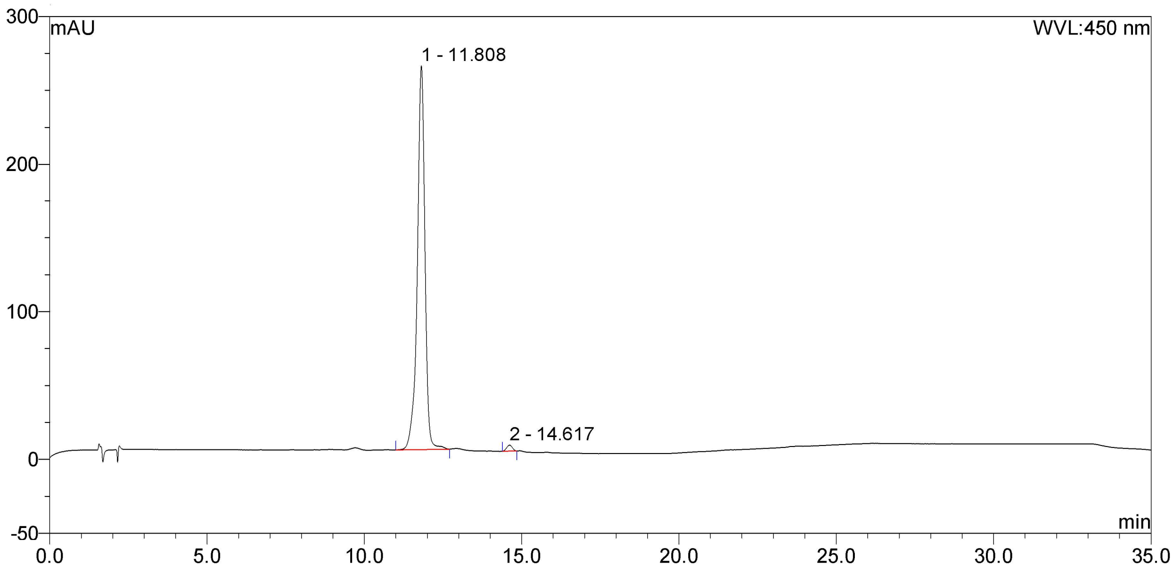

Figure S23. HPLC chromatogram of 8′-apo-β-carotenol monomelatonin (**18**) at 450 nm

**No.**	**Ret.Time min**	**Peak Name**	**Height mAU**	**Area mAU*min**	**Rel.Area %**	**Amount**	**Type**
1	11,81	n.a.	260,073	73,076	98,86	n.a.	BMB
2	14,62	n.a.	4,061	0,844	1,14	n.a.	BMB*
**Total:**			264,134	73,920	100,00	0,000	
